# Supplementary material for: A secreted metal-binding protein protects necrotrophic phytopathogens from reactive oxygen species
Source: Nat Commun. 2019 Oct 24;10:4853. doi: 10.1038/s41467-019-12826-x (PMC6813330; doi:10.1038/s41467-019-12826-x)
Supplement: Supplementary file 1 — Supplementary Information [file 41467_2019_12826_MOESM1_ESM.pdf]

Supplementary information

A secreted metal-binding protein protects necrotrophic phytopathogens from reactive oxygen species

Lulu Liu, Virginie Gueguen-Chaignon, Isabelle R Gonçalves, Christine Rascle, Martine Rigault, Alia Dellagi, Elise Loisel, Nathalie Poussereau, Agnès Rodrigue, Laurent Terradot, Guy Condemine

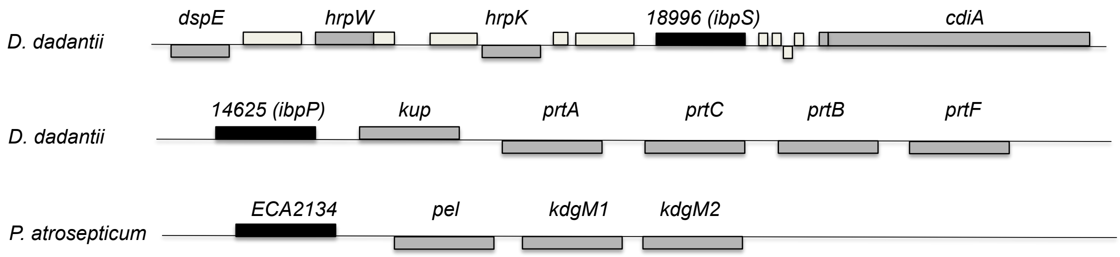

Supplementary Fig. 1 Genetic environment of *D. dadantii ibpS*, *ibpP* and *P. atrosepticum* ECA2134. The genes *ibpS*, *ibpP* and *ECA2134* are shown as black boxes. Genes with known functions are shown in dark grey, and those with unknown function are shown in light grey. *D. dadantii* 3937 GenBank accession no. CP002038.1, *P. atrosepticum* GenBank accession no. BX950851.1.

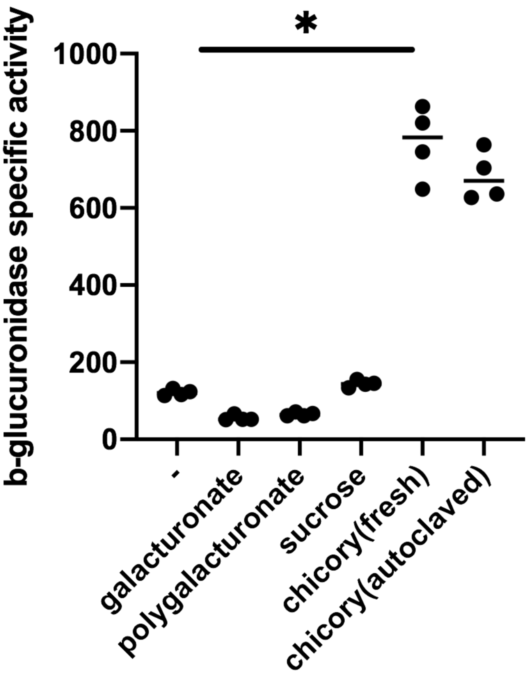

Supplementary Fig. 2 Expression of *D. dadantii ibpS* in various growth conditions. *D. dadantii* strain A5488 containing the *ibpS-uidA* fusion was grown in M63 + glycerol medium in the presence of the indicated compounds. ß-glucuronidase activity was measured with *p*-nitrophenyl-ß-D-glucuronate. Activities are expressed in µmoles of *p*-nitrophenol produced per minute and per milligram of bacterial dry weight ± standard deviation. Data are expressed as the mean (n = 4) from four independent experiments. * denotes a significant difference $p < 0.05$ two-sided Mann-Whitney test.

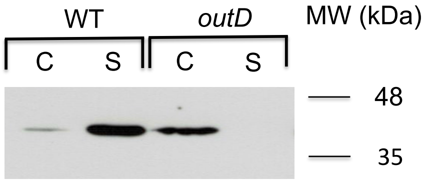

Supplementary Fig. 3 *D. dadantii* IbpS is secreted by the Out secretion system. Wild-type and *outD* mutant strains were grown overnight in M63 + glycerol medium containing a slice of chicory. The supernatant (S) and cellular (C) fractions were separated by SDS-PAGE. After blotting, IbpS was immunodetected.

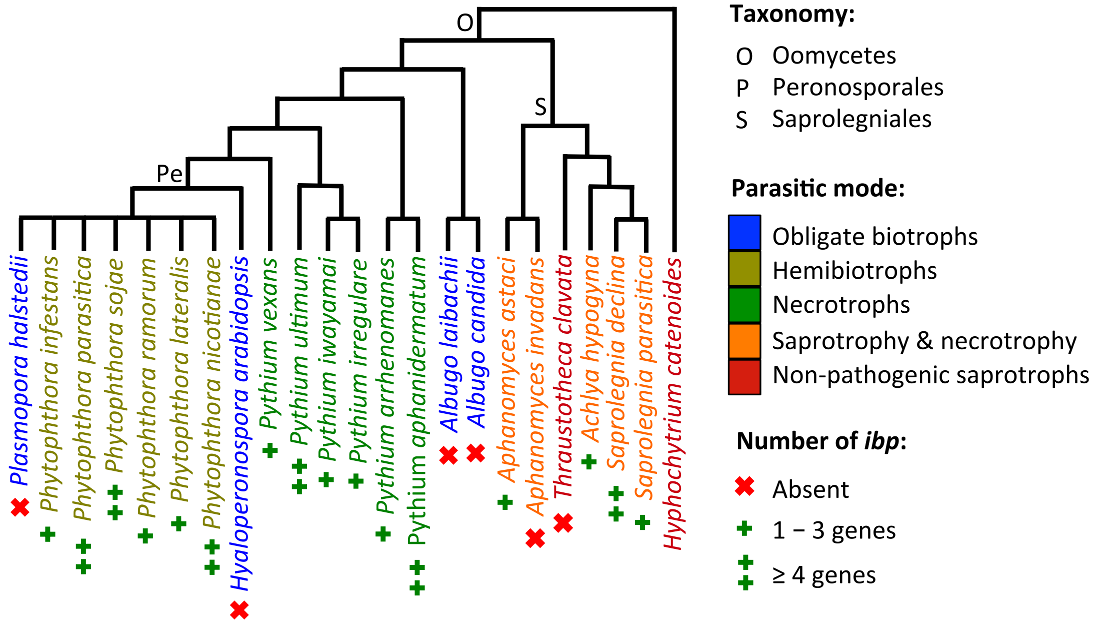

Supplementary Fig. 4 The number of *ibp* genes is associated with the parasitic mode in oomycetes. The schematic representation of the phylogeny of the oomycetes with *Hyphochytrium catenoides* as an outgroup is adapted from [1]. *ibp* gene numbers results from the PSI-BLAST search with the NCBI nr database (see Methods) and additional BLASTP searches on the EnsemblProtist website.

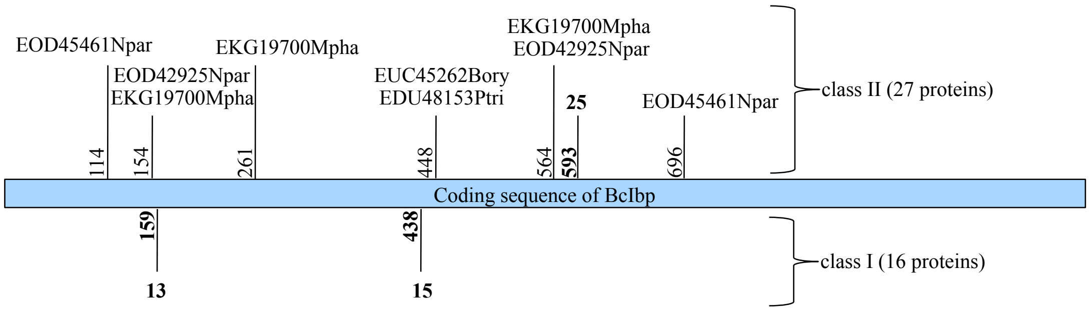

Supplementary Fig. 5 Fungal class I and II Ibp-encoding genes have no common introns. The intron positions were mapped on the coding sequence (CDS) of the *Botrytis cinerea ibp* gene, after alignment of the *ibp* CDSs of the two classes from the corresponding multiple protein alignment (http://wwwabi.snv.jussieu.fr/public/Clustal2Dna/). Each vertical line represents an observed intron position, written on the side of these lines. At their top (class II) or bottom (class I) tip, the number of protein sequences having the corresponding intron is marked or replaced with the name of the corresponding. For the list of genes belonging to classes I and class II, see supplementary Data 2.

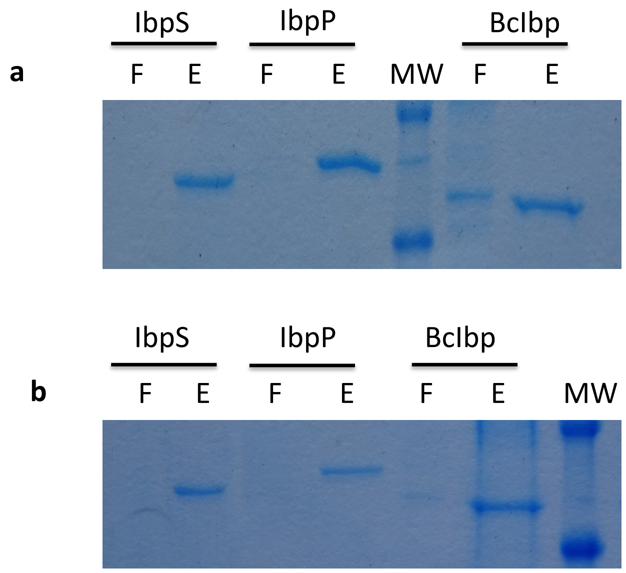

Supplementary Fig. 6 IbpS, IbpP and BcIbp bind iron and copper. **a-b** IbpS, IbpP or BcIbp (10 µg) in 100 µl of A buffer was incubated with 100 µl of the (**a**) Cu-NTA or (**b**) Fe-NTA resins for 15 min. After centrifugation the supernatants (flowthroughs) were removed and the resins were washed three times with 1 ml of buffer A. Protein was eluted with 100 µl of 50 mM EDTA and 10 µl of the flowthrough (F) or eluate (E) was loaded onto a SDS-PAGE gel.

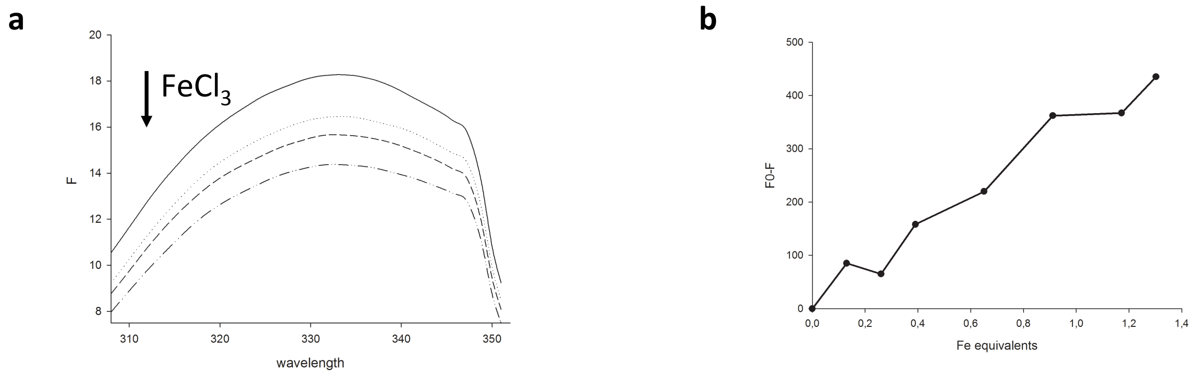

Supplementary Fig. 7 a Fluorescence spectra of 70 µM IbpS titrated with up to 10 equivalents of $FeCl_3$ added sequentially. **b** Fluorescence spectra of 38 µM IbpS titrated with the indicated equivalents of $FeCl_3$. The F0-F values are plotted.

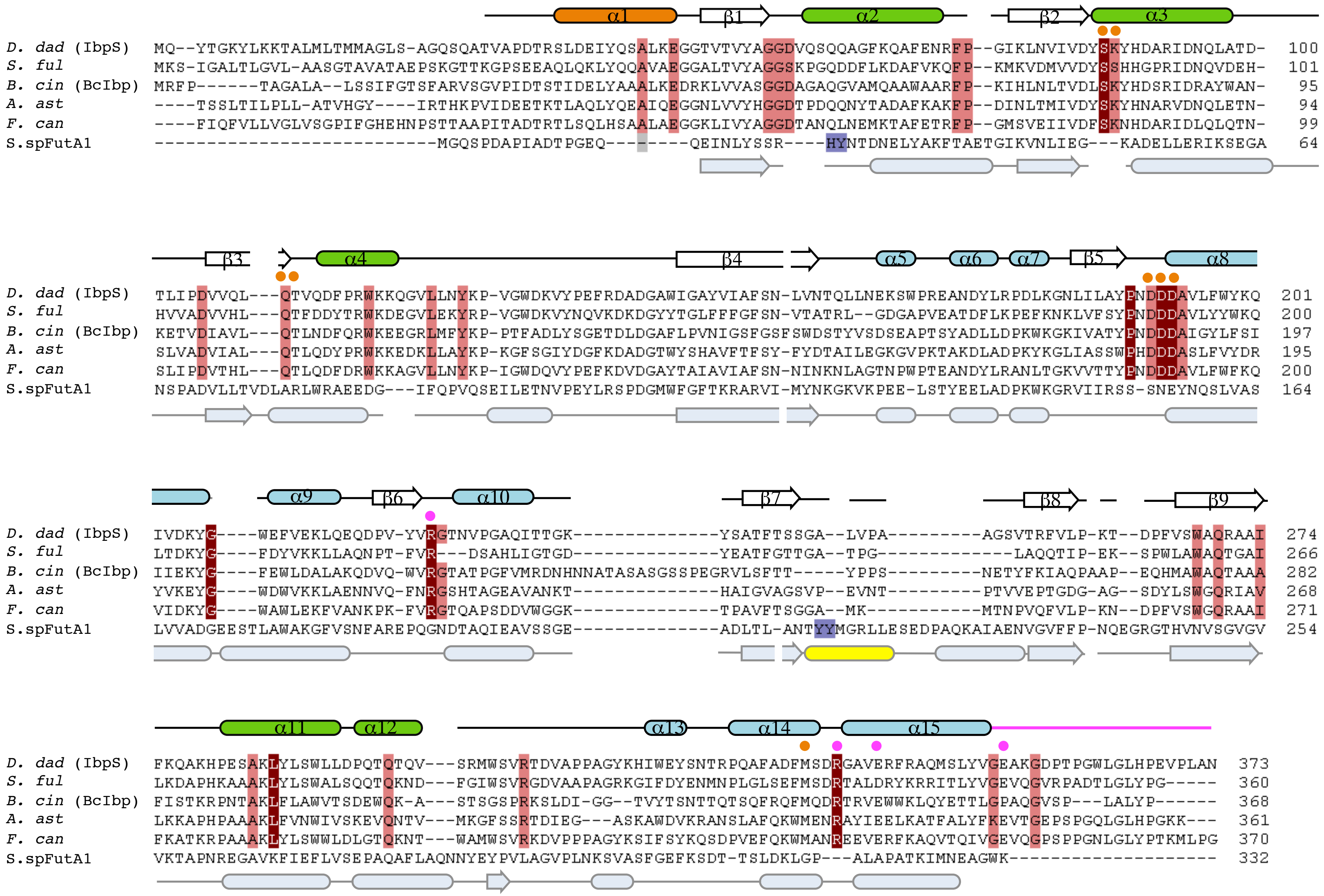
α1 β1 α2 β2 α3
D. dad (IbpS) MQ--YTGKYLKKTALMLTMMAGLS-AGQSQATVAPDTRSLDEIYQSALKEGGTVTVYAGGDVQSQQAGFKQAFENRFP--GIKLNVIVDYSKYHDARIDNQLATD- 100
S. ful MKS-IGALTLGVL-AASGTAVATAEPSKGTTKGPSEEAQLQKLYQQAVAEGGALTVYAGGSKPGQDDFLKDAFVKQFP--KMKVDMVVDYSSHHGPRIDNQVDEH- 101
B. cin (BcIbp) MRFP------TAGALA--LSSIFGTSFARVSGVPIDTSTIDELYAAALKEDRKLVVASGGDAGAQGVAMQAAWAARFP--KIHLNLTVDLSKYHDSRIDRAYWAN- 95
A. ast ----TSSLTILPLL-ATVHGY----IRTHKPVIDEETKTLAQLYQEAIQEGGNLVVYHGGDTPDQQNYTADAFKAKFP--DINLTMIVDYSKYHNARVDNQLETN- 94
F. can ----FIQFVLLVGLVSGPIFGHEHNPSTTAAPITADTRTLSQLHSAALAEGGKLIVYAGGDTANQLNEMKTAFETRFP--GMSVEIIVDFSKNHDARIDLQLQTN- 99
S.spFutA1 ---------------------------MGQSPDAPIADTPGEQ---------QEINLYSSR----HYNTDNELYAKFTAETGIKVNLIEG---KADELLERIKSEGA 64
β3 α4 β4 α5 α6 α7 β5 α8
D. dad (IbpS) TLIPDVVQL---QTVQDFPRWKKQGVLLNYKP-VGWDKVYPEFRDADGAWIGAYVIAFSN-LVNTQLLNEKSWPREANDYLRPDLKGNLILAYPNDDDAVLFWYKQ 201
S. ful HVVADVVHL---QTFDDYTRWKDEGVLEKYRP-VGWDKVYNQVKDKDGYYTGLFFFGFSN-VTATRL--GDGAPVEATDFLKPEFKNKLVFSYPNDDDAVLYYWKQ 200
B. cin (BcIbp) KETVDIAVL---QTLNDFQRWKEEGRLMFYKP-PTFADLYSGETDLDGAFLPVNIGSFGSFSWDSTYVSDSEAPTSYADLLDPKWKGKIVATYPNDDDAIGYLFSI 197
A. ast SLVADVIAL---QTLQDYPRWKKEDKLLAYKP-KGFSGIYDGFKDADGTWYSHAVFTFSY-FYDTAILEGKGVPKTAKDLADPKYKGLIASSWPHDDDASLFVYDR 195
F. can SLIPDVTHL---QTLQDFDRWKKAGVLLNYKP-IGWDQVYPEFKDVDGAYTAIAVIAFSN-NINKNLAGTNPWPTEANDYLRANLTGKVVTTYPNDDDAVLFWFKQ 200
S.spFutA1 NSPADVLLTVDLARLWRAEEDG---IFQPVQSEILETNVPEYLRSPDGMWFGFTKRARVI-MYNKGKVKPEE-LSTYEELADPKWKGRVIIRSS-SNEYNQSLVAS 164
α9 β6 α10 β7 β8 β9
D. dad (IbpS) IVDKYG----WEFVEKLQEQDPV-YVRGTNVPGAQITTGK--------------YSATFTSSGA--LVPA---------AGSVTRFVLP-KT--DPFVSWAQRAAI 274
S. ful LTDKYG----FDYVKKLLAQNPT-FVR---DSAHLIGTGD--------------YEATFGTTGA--TPG--------------LAQQTIP-EK--SPWLAWAQTGAI 266
B. cin (BcIbp) IIEKYG----FEWLDALAKQDVQ-WVRGTATPGFVMRDNHNNATASASGSSPEGRVLSFTT-----YPPS---------NETYFKIAQPAAP--EQHMAWAQTAAA 282
A. ast YVKEYG----WDWVKKLAENNVQ-FNRGSHTAGEAVANKT--------------HAIGVAGSVP--EVNT---------PTVVEPTGDG-AG--SDYLSWGQRIAV 268
F. can VIDKYG----WAWLEKFVANKPK-FVRGTQAPSDDVWGGK--------------TPAVFTSGGA--MK-----------MTNPVQFVLP-KN--DPFVSWGQRAAI 271
S.spFutA1 LVVADGEESTLAWAKGFVSNFAREPQGNDTAQIEAVSSGE--------------ADLTL-ANTYYMGRLLESEDPAQKAIAENVGVFFP-NQEGRGTHVNVSGVGV 254
α11 α12 α13 α14 α15
D. dad (IbpS) FKQAKHPESAKLYLSWLLDPQTQTQV---SRMWSVRTDVAPPAGYKHIWEYSNTRPQAFADFMSDRGAVERFRAQMSLYVGEAKGDPTPGWLGLHPEVPLAN 373
S. ful LKDAPHKAAAKLYLSWALSQQTQKND---FGIWSVRGDVAAPAGRKGIFDYENMNPLGLSEFMSDRTALDRYKRRITLYVGEVQGVRPADTLGLYPG----- 360
B. cin (BcIbp) FISTKRPNTAKLFLAWVTSDEWQK-A---STSGSPRKSLDI-GG--TVYTSNTTQTSQFRQFMQDRTRVEWWKLQYETTLGPAQGVSP---LALYP------ 368
A. ast LKKAPHPAAAKLFVNWIVSKEVQNTV---MKGFSSRTDIEG---ASKAWDVKRANSLAFQKWMENRAYIEELKATFALYFKEVTGEPSPGQLGLHPGKK--- 361
F. can FKATKRPAAAKLYLSWWLDLGTQKNT---WAMWSVRKDVPPPAGYKSIFSYKQSDPVEFQKWMANREEVERFKAQVTQIVGEVQGPSPPGNLGLYPTKMLPG 370
S.spFutA1 VKTAPNREGAVKFIEFLVSEPAQAFLAQNNYEYPVLAGVPLNKSVASFGEFKSDT-TSLDKLGP---ALAPATKIMNEAGWK------------------- 332

Supplementary Fig. 8. Structure-based sequence alignment of IbpS with homologs. Sequences corresponding to Ibp proteins from *Dickeya dadantii* (IbpS, ABF18996), *Streptomyces fulvoviolaceus* (WP030598511), *Botrytis cinerea* (BcIbp, 09p00460), *Aphanomyces astaci* (ETV75801), *Folsomia candida* (XP029143086) were aligned with Clustal O. The corresponding species are also indicated in the phylogenetic tree (Fig. 1). The sequence of FutA1 from *Synechocystis sp.* corresponding to the crystal structure (pdb 3F11) was aligned by structural superimposition using DALI. Residues coordinating iron in FutA1 are shaded in blue. Secondary structures of IbpS and of FutA1 are indicated above and below the sequences, respectively and coloured according to Fig. 3a. Strictly conserved residues are coloured in firebrick and strongly conserved (95%) residues are coloured in salmon. Orange dots indicate residues participating to the iron-binding pocket in IbpS. Magenta dots indicate residues involved in the dimerisation interface.

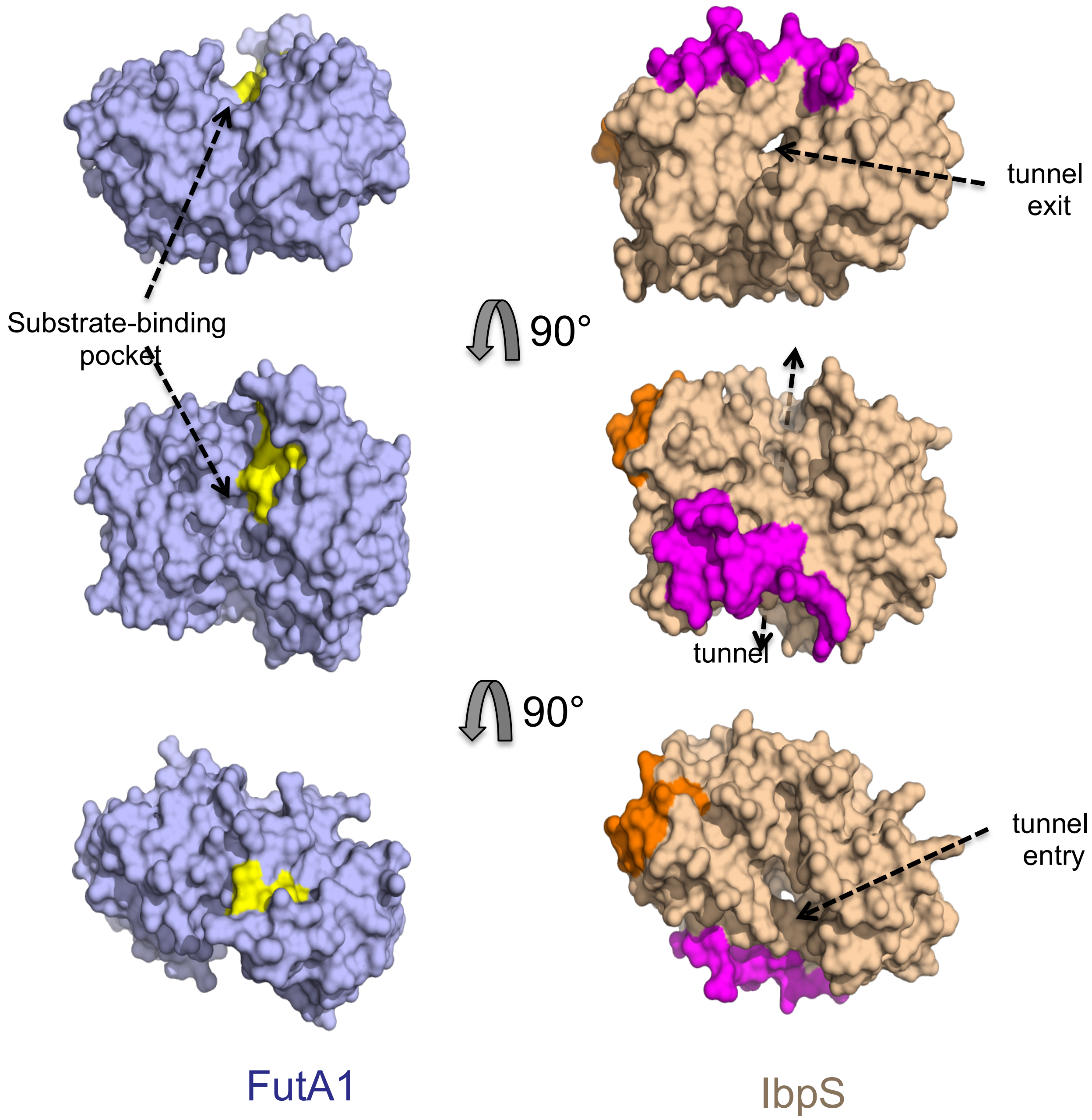

Supplementary Fig. 9. Structural comparison of the accessible surfaces in FutA1 (pdb code 3F11) and IbpS structures. The structures are coloured in blue (FutA1) with the helix involved in iron binding coloured in yellow as in Fig 3a). IbpS is coloured in wheat, except for the additional N-terminal helix (orange) and C-terminal tail (magenta). The figure illustrates the structural difference of the canonical substrate-binding pocket and the presence of a tunnel in IbpS structure.

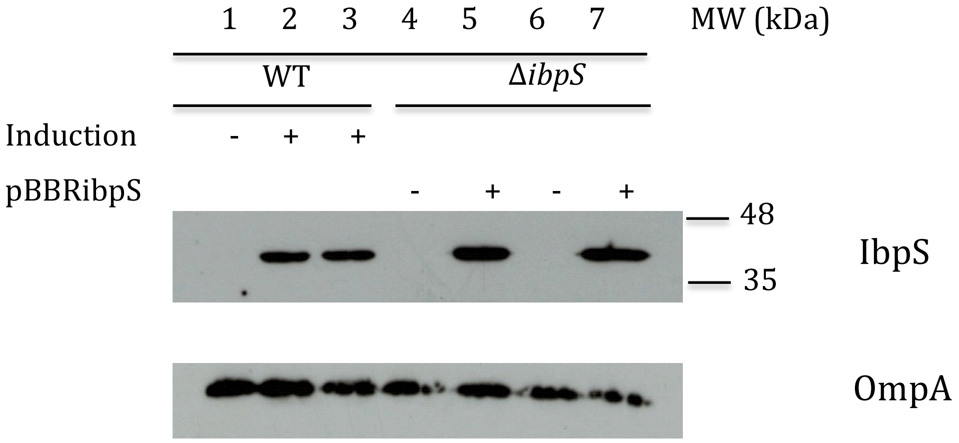

Supplementary Fig. 10. Quantification of IbpS produced by different strains. IbpS was quantified using IbpS antibodies in 15 μl of culture supernatant of the wild type strain A4922 without (lane 1) or with (lane 2 and 3) induction by chicory and the *ibpS* mutant A5488 containing the empty plasmid pBBRmcs5 (lane 4 and 6) or plasmid pBBRibpS (lane 5 and 7). OmpA antibody was used as a loading control.

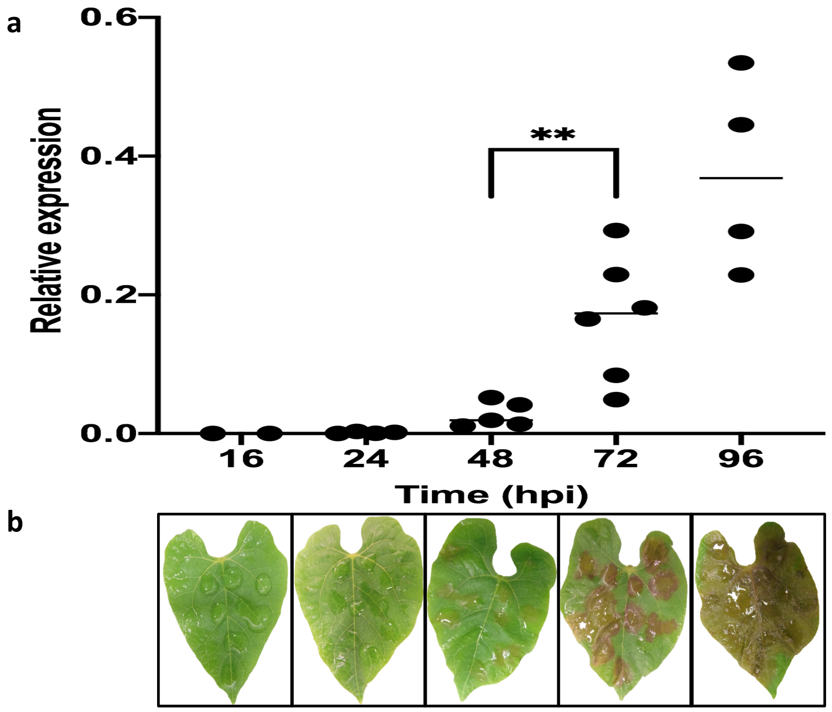

Supplementary Fig. 11 The *Bcibp* gene is expressed by *B. cinerea* during infection. **a** Expression levels of the *BcIbp* gene during the kinetics of French bean leaf infection by *B. cinerea*. The actin-encoding gene, the *bcef1α* gene (Bcin09g05760) and the *bcpda1* gene (Bcin07g01890) were used as a reference. Three independent biological replicates were assessed for each experiment. Standard deviations are indicated, and the asterisks indicate a significant difference (Mann and Whitney test, ** *p*-value <0.005) in gene expression compared with that at a previous time point. **b** The stages of infection are shown for each time point.

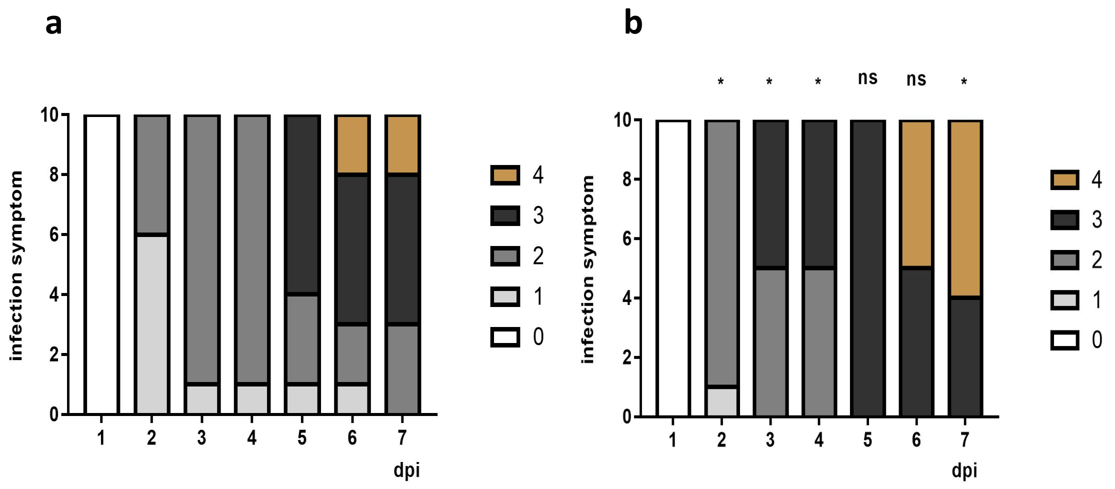

Supplementary Fig. 12 Complementation of the *ibpS* mutation. Evolution of the symptoms on *A. thaliana* Col0 inoculated by **a** the *ibpS* mutant strain containing the empty plasmid pBBRmcs5 and **b** the complemented *ibpS*/pBBRibpS strain. Infection was performed on a single leaf by the deposition of a drop containing approximately 50 bacteria on a wound made by a needle. Symptoms were classified in five stages as shown on the right. stage 0: no symptoms; stage 1, symptoms around the spot of infection: stage 2: maceration of the leaf limb; stage 3: maceration of the whole leaf, including the petiole; stage 4: generalization to the whole plant. * indicates a statistical difference ($p < 0.05$) between results of the *ibpS*/pBBRmcs5 and *ibpS*/pBBRibpS strains at a given day. ns: not significant.

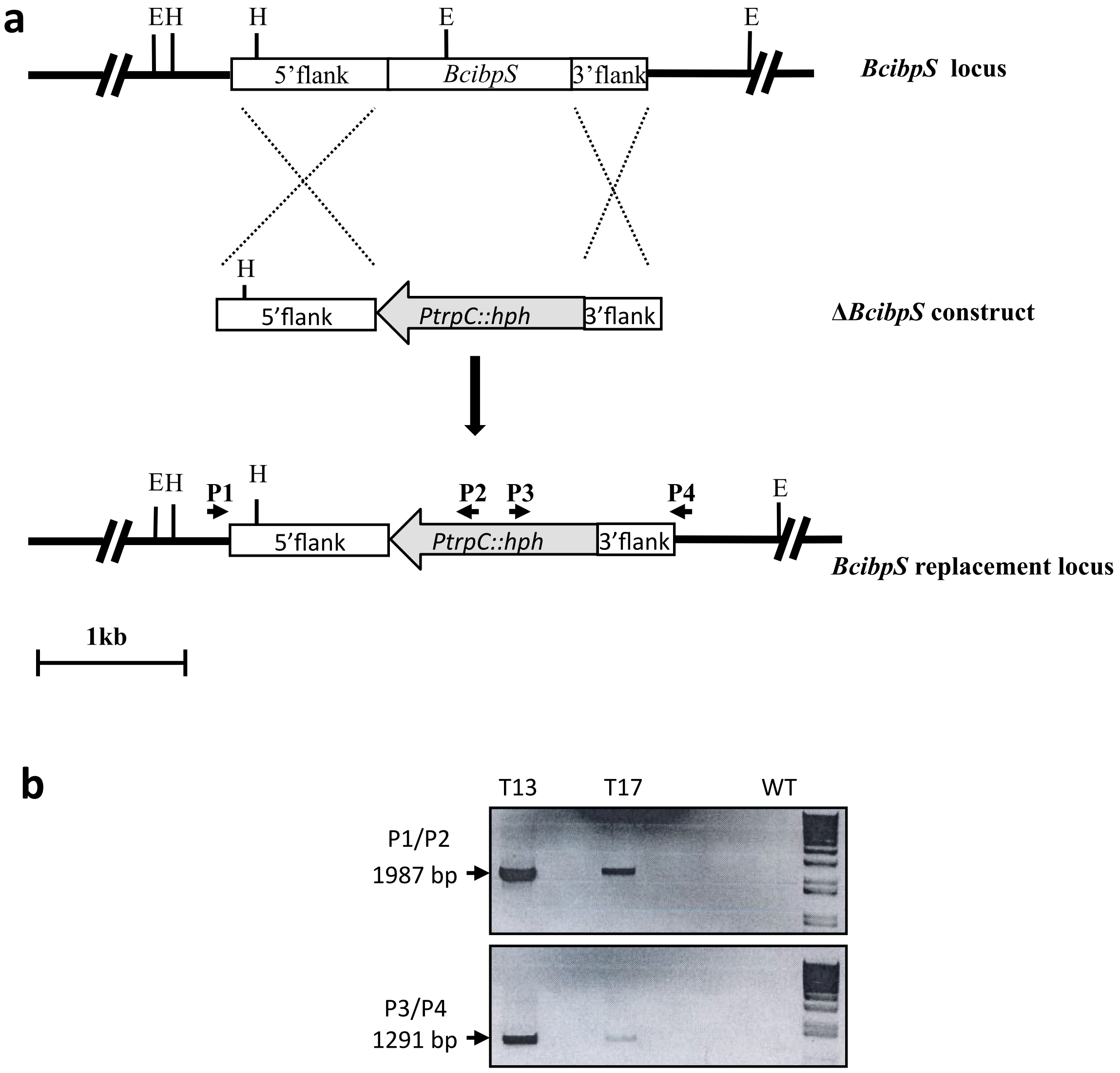

Supplementary Fig. 13 Construction and verification of *B. cinerea Bcibp*-null mutants. **a** Schematic representation of replacement of the *Bcibp* gene with the hygromycin resistance gene (*hph*) flanked by 1.080 kp of 5' sequence and 0.522 kb of 3' sequence from the *Bcibp* locus. The primers (black arrows) used for PCR analysis are indicated. **b** Diagnostic PCR was performed to identify the deletion mutants. The primers pairs are indicated.

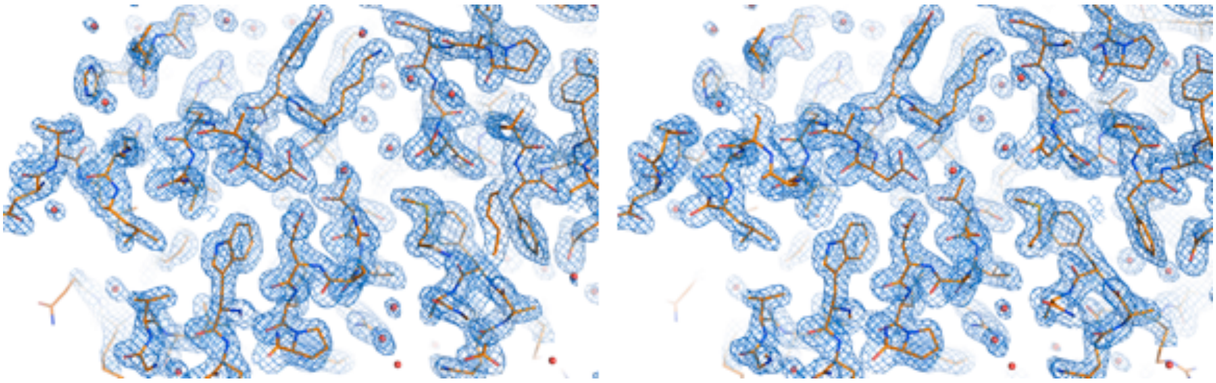

Supplementary Fig. 14 Stereo view of the 2Fo-Fc density map of IbpS crystal structure countoured at 1.2 σ. IbpS model is depicted as ball and stick (carbone, orange ; oxygene, red ; nitrogen, blue) and water molecules are represented as red spheres.

Supplementary Table 1 Strains used in this study

Strains	Description	Reference
D. dadantii		
A4922	Wild type 3937	Laboratory collection
A5488	*ibpS*::*uidA*-kan	This work
A5653	*outD::*Cm^R	Laboratory collection
A6050	*acsA*::*lacZ*-kan	[2]
E. coli		
NM522	Δ(*lac-proAB*) *thi hsd*-5 *supE (*F' *proAB lacI*^{q} Δ*lacZ*M15)	Stratagene
W3110	Wild type	Laboratory collection
WczcR3	W3110/pczcR3	This work
**Plasmids**		
pczcR3	*czcR3*-P*czcR3*::*lux* in plux Kan^R	This work
pBBRmcs5	Gm^R	[3]
pBBRibpS	pBBRmcs5 containing *ibpS*	This work

Supplementary Table 2 Oligonucleotides used in this study

18996L+	gatcagcacgctctcttccg
18996L-	gtaatggttgctccagttcgc
14625+	gctatcggcccttaacgcac
14625-	cggataacttagtgcggcag
18996GEX+	ccgaattccactgtcgcgccggacacccg
18996GEX-	cgctcgagttagttcgccagcggcacctc
14625GEX+	cccggatccgccatcacgccggaacagcg
14625 GEX-	cgcctcgagttagtgcggcagcgcctg
Bc96GEX+	cccggatccgttccaatcgatacctcgac
Bc96GEX-	gggctcagattaagggtacaaagccaaag
bHLH38 Forward	taccgacgcaagaagatcaac
bHLH38 Reverse	tccttgagatcttgatgatgaaac
AtFER1 Forward	cagactacatcacccagctaagga
AtFER1 Reverse	tctggtcgaaatgccaaactc
Clathrin Forward	gtttgggagaagagcggtta
Clathrin Reverse	ctgatgtcactgaacctgaactg
For-ATG	tttcacaagcttgaattcatgcgtttcccgactgcc
Rev-TAA	tttcacaagcttgaattcttaagggtacaaagccaaaggacta
bcibpS-For	cagcagccccggaaca
bcibpS-Rev	gaaataaaagccgcagctgtct
bcactA-For	ccgtgctccagaagctttgt
bcactA-Rev	gtggataccaccgctctcaag
For-5'-ibp	ccgcagcattctaagtctagct
Rev-5'-ibp	atgccgaccgggaacggatcgagatgaagcaggaa
For-3'-ibp	agggcctgagtggccgttctcaggggagctagtggg
Rev-3'-ibp	gaaattggctttgcacaagga
For-Hygro	attcgagggcctgagtggcc
Rev-Hygro	gttcccggtcggcatctact
PCR3-For	gggttccacattccaatgtcc
PCR3-Rev	cagatggggcaaaggtcttc
P1	ccgcagcattctaagtctagct
P2	cattggggagttcagcgagagcc
P3	ggctctcgctgaactccccaatg
P4	gaaattggctttgcacaagga
PP2a3-F	gcaatctctcattccgatagtc
PP2a3-R	ataccgaacatcaacatctgg
bcef1a-F	ttcccgcccaactgaca
bcef1a-R	cgttccaataccaccaatcttgt
bcpda1-F	cgctgttaaggctgctgtca
bcpda1-R	cgaggactaatggaccgttacc

Supplementary references

1. Savory F, Leonard G, Richards TA. The role of horizontal gene transfer in the evolution of the oomycetes. *PLoS Pathog* **11**, e1004805 (2015).

2. Franza T, Mahe B, Expert D. *Erwinia chrysanthemi* requires a second iron transport route dependent of the siderophore achromobactin for extracellular growth and plant infection. *Mol Microbiol* **55**, 261-275 (2005).

3. Obranic S, Babic F, Maravic-Vlahovicek G. Improvement of pBBR1MCS plasmids, a very useful series of broad-host-range cloning vectors. *Plasmid* **70**, 263-267 (2013).
