# Supplementary material for: A secreted metal-binding protein protects necrotrophic phytopathogens from reactive oxygen species
Source: Nat Commun. 2019 Oct 24;10:4853. doi: 10.1038/s41467-019-12826-x (PMC6813330; doi:10.1038/s41467-019-12826-x)
Supplement: Supplementary file 3 — Description of Additional Supplementary Files [file 41467_2019_12826_MOESM3_ESM.pdf]

Description of Additional Supplementary Files

File Name: Supplementary Data 1
Description: Supplementary Data 1 summarizes the results of the bioinformatic search of Ibp proteins in the NCBI non-redundant (nr) database. The file contains the accession numbers of 898 putative Ibp proteins between brackets, with the taxonomy of the corresponding 222 bacteria and 162 eukaryotes.

File Name: Supplementary Data 2
Description: Supplementary Data 2 summarizes characteristics of the 122 putative Ibp proteins, chosen for the phylogeny. Worksheets contain the name, accession numbers, class where appropriate, and bioinformatic predictions of signal sequences for putative Ibp proteins of 34 bacteria, 30 fungi, 5 oomycetes and 2 metazoa. Other worksheets contain the taxonomy of the corresponding species, their number of putative Ibp-encoding genes, and the Gram stain for bacteria.
