# Supplementary material for: A secreted metal-binding protein protects necrotrophic phytopathogens from reactive oxygen species
Source: Nat Commun. 2019 Oct 24;10:4853. doi: 10.1038/s41467-019-12826-x (PMC6813330; doi:10.1038/s41467-019-12826-x)
Supplement: Supplementary file 4 — Supplementary Data 1 [file 41467_2019_12826_MOESM4_ESM.pdf]

Supplementary Data1 List of species, in the NCBI nonredundant (nr) database containing at least one protein from the Ibp family

```
# NCBI taxonomy [protein accesion number(s)]
Bacteria; Actinobacteria; Actinobacteria bacterium OK074 [WP_054212245.1 WP_054212889.1 ]
Bacteria; Actinobacteria; Actinobacteria bacterium OV320 [WP_054241162.1 ]
Bacteria; Actinobacteria; Micrococcales; Microbacteriaceae; Agromyces; Agromyces sp. Soil535 [WP_056731231.1 ]
Bacteria; Actinobacteria; Micrococcales; Microbacteriaceae; Mycetocola; Mycetocola reblochoni [WP_087137757.1 ]
Bacteria; Actinobacteria; Micromonosporales; Micromonosporaceae; Actinoplanes; Actinoplanes subtropicus [WP_030435168.1
WP_030437413.1 ]
Bacteria; Actinobacteria; Pseudonocardiales; Pseudonocardiaceae; Alloactinosynnema sp. L-07 [WP_054048703.1 ]
Bacteria; Actinobacteria; Pseudonocardiales; Pseudonocardiaceae; Amycolatopsis; Amycolatopsis balhimycina [WP_020639576.1 ]
Bacteria; Actinobacteria; Pseudonocardiales; Pseudonocardiaceae; Amycolatopsis; Amycolatopsis mediterranei [WP_013227324.1 ]
Bacteria; Actinobacteria; Pseudonocardiales; Pseudonocardiaceae; Amycolatopsis; Amycolatopsis tolypomycina [SED61397.1 ]
Bacteria; Actinobacteria; Pseudonocardiales; Pseudonocardiaceae; Crossiella; Crossiella equi [WP_086788139.1 ]
Bacteria; Actinobacteria; Pseudonocardiales; Pseudonocardiaceae; Lechevalieria; Lechevalieria aerocolonigenes [WP_082115452.1
KJK34673.1 ]
Bacteria; Actinobacteria; Pseudonocardiales; Pseudonocardiaceae; Lentzea; Lentzea kentuckyensis [WP_086668024.1 ]
Bacteria; Actinobacteria; Pseudonocardiales; Pseudonocardiaceae; Pseudonocardia; Pseudonocardia sp. CNS-004 [WP_075952647.1 ]
Bacteria; Actinobacteria; Streptomycetales; Streptomycetaceae; Streptomyces [WP_006382038.1 WP_006382519.1 WP_044473499.1
WP_031057213.1 WP_060882300.1 WP_031135472.1 WP_079158572.1 WP_031045817.1 ]
Bacteria; Actinobacteria; Streptomycetales; Streptomycetaceae; Kitasatospora; Kitasatospora setae [WP_014135001.1 ]
Bacteria; Actinobacteria; Streptomycetales; Streptomycetaceae; Streptomyces; Actinosporangium sp. NRRL B-3428 [WP_052582889.1 ]
Bacteria; Actinobacteria; Streptomycetales; Streptomycetaceae; Streptomyces; Streptomyces acidiscabies [WP_059042154.1
WP_010359574.1 WP_075730936.1 WP_050373746.1 WP_010352795.1 WP_050374021.1 WP_029184289.1 WP_050370202.1 WP_010355847.1
WP_059044648.1 WP_075737047.1 WP_075738186.1 WP_063870985.1 KND40201.1 WP_078841121.1 WP_075779477.1 WP_010355164.1 WP_050373317.1
WP_075736773.1 WP_059043037.1 ]
Bacteria; Actinobacteria; Streptomycetales; Streptomycetaceae; Streptomyces; Streptomyces aidingensis [SFD09714.1 ]
Bacteria; Actinobacteria; Streptomycetales; Streptomycetaceae; Streptomyces; Streptomyces albireticuli [WP_087924720.1 ]
Bacteria; Actinobacteria; Streptomycetales; Streptomycetaceae; Streptomyces; Streptomyces alboverticillatus [WP_086571414.1 ]
Bacteria; Actinobacteria; Streptomycetales; Streptomycetaceae; Streptomyces; Streptomyces antibioticus [WP_059190966.1
WP_059190329.1 ]
Bacteria; Actinobacteria; Streptomycetales; Streptomycetaceae; Streptomyces; Streptomyces bobili [WP_086773076.1 ]
Bacteria; Actinobacteria; Streptomycetales; Streptomycetaceae; Streptomyces; Streptomyces bottropensis [WP_005484046.1
WP_005485028.1 ]
Bacteria; Actinobacteria; Streptomycetales; Streptomycetaceae; Streptomyces; Streptomyces caatingaensis [WP_049717969.1 ]
Bacteria; Actinobacteria; Streptomycetales; Streptomycetaceae; Streptomyces; Streptomyces caeruleatus [WP_062716822.1 ]
Bacteria; Actinobacteria; Streptomycetales; Streptomycetaceae; Streptomyces; Streptomyces canus [WP_059212102.1 WP_059211066.1 ]
Bacteria; Actinobacteria; Streptomycetales; Streptomycetaceae; Streptomyces; Streptomyces cellulosae [WP_030674478.1 ]
Bacteria; Actinobacteria; Streptomycetales; Streptomycetaceae; Streptomyces; Streptomyces chartreusis [WP_010048360.1 ]
Bacteria; Actinobacteria; Streptomycetales; Streptomycetaceae; Streptomyces; Streptomyces ciscaucasicus [WP_062050312.1 ]
Bacteria; Actinobacteria; Streptomycetales; Streptomycetaceae; Streptomyces; Streptomyces europaeiscabiei [WP_046703142.1
WP_060893840.1 WP_046707323.1 WP_060890227.1 WP_046708110.1 WP_063820122.1 ]
Bacteria; Actinobacteria; Streptomycetales; Streptomycetaceae; Streptomyces; Streptomyces fulvoviolaceus [WP_030612287.1
WP_030611286.1 WP_030598511.1 ]
Bacteria; Actinobacteria; Streptomycetales; Streptomycetaceae; Streptomyces; Streptomyces galbus [WP_033525254.1 ]
Bacteria; Actinobacteria; Streptomycetales; Streptomycetaceae; Streptomyces; Streptomyces griseoruber [WP_059202568.1
WP_079109097.1 WP_055633632.1 ]
```

Bacteria; Actinobacteria; Streptomycetales; Streptomycetaceae; Streptomyces; Streptomyces griseorubiginosus [WP_062244424.1]
Bacteria; Actinobacteria; Streptomycetales; Streptomycetaceae; Streptomyces; Streptomyces hokutonensis [WP_019074706.1 WP_019074553.1 WP_019075643.1]
Bacteria; Actinobacteria; Streptomycetales; Streptomycetaceae; Streptomyces; Streptomyces humi [WP_046733713.1]
Bacteria; Actinobacteria; Streptomycetales; Streptomycetaceae; Streptomyces; Streptomyces ipomoeae [WP_009329103.1]
Bacteria; Actinobacteria; Streptomycetales; Streptomycetaceae; Streptomyces; Streptomyces ipomoeae 91-03 [EKX61718.1]
Bacteria; Actinobacteria; Streptomycetales; Streptomycetaceae; Streptomyces; Streptomyces lincolnensis [WP_067427093.1]
Bacteria; Actinobacteria; Streptomycetales; Streptomycetaceae; Streptomyces; Streptomyces mirabilis [WP_037722053.1 WP_037749412.1 WP_075032088.1 WP_037712805.1 WP_037713639.1 WP_078939848.1]
Bacteria; Actinobacteria; Streptomycetales; Streptomycetaceae; Streptomyces; Streptomyces mobaraensis [WP_004940520.1]
Bacteria; Actinobacteria; Streptomycetales; Streptomycetaceae; Streptomyces; Streptomyces neyagawaensis [WP_055542606.1]
Bacteria; Actinobacteria; Streptomycetales; Streptomycetaceae; Streptomyces; Streptomyces niveiscabiei [WP_055721667.1 WP_055724175.1 WP_055724162.1 WP_055721903.1 WP_055724101.1]
Bacteria; Actinobacteria; Streptomycetales; Streptomycetaceae; Streptomyces; Streptomyces ossamyceticus [WP_055517828.1]
Bacteria; Actinobacteria; Streptomycetales; Streptomycetaceae; Streptomyces; Streptomyces phaeochromogenes [WP_055616915.1]
Bacteria; Actinobacteria; Streptomycetales; Streptomycetaceae; Streptomyces; Streptomyces phaeopurpureus [WP_062030996.1]
Bacteria; Actinobacteria; Streptomycetales; Streptomycetaceae; Streptomyces; Streptomyces prunicolor [WP_026150399.1]
Bacteria; Actinobacteria; Streptomycetales; Streptomycetaceae; Streptomyces; Streptomyces regalis [WP_062704812.1 WP_062711466.1]
Bacteria; Actinobacteria; Streptomycetales; Streptomycetaceae; Streptomyces; Streptomyces resistomycificus [WP_030044319.1 WP_030044322.1]
Bacteria; Actinobacteria; Streptomycetales; Streptomycetaceae; Streptomyces; Streptomyces reticuliscabiei [WP_086810346.1]
Bacteria; Actinobacteria; Streptomycetales; Streptomycetaceae; Streptomyces; Streptomyces scabiei [WP_086746256.1 WP_059081748.1 WP_086757490.1 WP_086785271.1 WP_086759264.1 WP_013006044.1 WP_037698553.1 WP_086799620.1 WP_060907729.1 WP_059078436.1 WP_046773362.1 WP_012998807.1 WP_086747130.1 WP_063817352.1 WP_060889484.1 WP_060888825.1 WP_061334752.1 WP_060881080.1 WP_060905855.1 WP_086797265.1 WP_037729275.1 WP_086759536.1 KFF98328.1]
Bacteria; Actinobacteria; Streptomycetales; Streptomycetaceae; Streptomyces; Streptomyces sp. 303MFCol5.2 [WP_020127576.1]
Bacteria; Actinobacteria; Streptomycetales; Streptomycetaceae; Streptomyces; Streptomyces sp. 3124.6 [WP_079663972.1]
Bacteria; Actinobacteria; Streptomycetales; Streptomycetaceae; Streptomyces; Streptomyces sp. 3213 [SEC36331.1 SEC37472.1 SEC39315.1]
Bacteria; Actinobacteria; Streptomycetales; Streptomycetaceae; Streptomyces; Streptomyces sp. 351MFTsu5.1 [WP_020133936.1]
Bacteria; Actinobacteria; Streptomycetales; Streptomycetaceae; Streptomyces; Streptomyces sp. 96-12 [WP_060882482.1]
Bacteria; Actinobacteria; Streptomycetales; Streptomycetaceae; Streptomyces; Streptomyces sp. AS58 [WP_053763589.1]
Bacteria; Actinobacteria; Streptomycetales; Streptomycetaceae; Streptomyces; Streptomyces sp. CdTB01 [WP_058920868.1]
Bacteria; Actinobacteria; Streptomycetales; Streptomycetaceae; Streptomyces; Streptomyces sp. DSM 15324 [WP_067254517.1]
Bacteria; Actinobacteria; Streptomycetales; Streptomycetaceae; Streptomyces; Streptomyces sp. FxanaA7 [WP_045560066.1 WP_045562120.1 WP_078853286.1]
Bacteria; Actinobacteria; Streptomycetales; Streptomycetaceae; Streptomyces; Streptomyces sp. LUP30 [WP_069767083.1 WP_069767917.1]
Bacteria; Actinobacteria; Streptomycetales; Streptomycetaceae; Streptomyces; Streptomyces sp. LUP47B [WP_069758130.1 WP_069760279.1]
Bacteria; Actinobacteria; Streptomycetales; Streptomycetaceae; Streptomyces; Streptomyces sp. MBT76 [WP_058043547.1]
Bacteria; Actinobacteria; Streptomycetales; Streptomycetaceae; Streptomyces; Streptomyces sp. NBRC 110027 [WP_042160067.1 GAO11530.1]
Bacteria; Actinobacteria; Streptomycetales; Streptomycetaceae; Streptomyces; Streptomyces sp. NRRL B-3229 [WP_030320164.1]
Bacteria; Actinobacteria; Streptomycetales; Streptomycetaceae; Streptomyces; Streptomyces sp. NRRL F-525 [WP_033279675.1]
Bacteria; Actinobacteria; Streptomycetales; Streptomycetaceae; Streptomyces; Streptomyces sp. NRRL S-646 [WP_030926834.1 WP_078912311.1]
Bacteria; Actinobacteria; Streptomycetales; Streptomycetaceae; Streptomyces; Streptomyces sp. NRRL WC-3618 [WP_053743357.1 WP_053740034.1 KOV88495.1]
Bacteria; Actinobacteria; Streptomycetales; Streptomycetaceae; Streptomyces; Streptomyces sp. Root264 [WP_057577066.1]

Bacteria; Actinobacteria; Streptomycetales; Streptomycetaceae; Streptomyces; Streptomyces sp. Root369 [WP_057613591.1]
Bacteria; Actinobacteria; Streptomycetales; Streptomycetaceae; Streptomyces; Streptomyces sp. S10(2016) [WP_062924668.1 WP_062927621.1]
Bacteria; Actinobacteria; Streptomycetales; Streptomycetaceae; Streptomyces; Streptomyces sp. WM6386 [WP_046258668.1]
Bacteria; Actinobacteria; Streptomycetales; Streptomycetaceae; Streptomyces; Streptomyces sp. cf124 [SFM90208.1]
Bacteria; Actinobacteria; Streptomycetales; Streptomycetaceae; Streptomyces; Streptomyces sp. cf386 [SDP79842.1]
Bacteria; Actinobacteria; Streptomycetales; Streptomycetaceae; Streptomyces; Streptomyces sp. yr375 [SEP68748.1]
Bacteria; Actinobacteria; Streptomycetales; Streptomycetaceae; Streptomyces; Streptomyces stelliscabiei [WP_046916510.1]
Bacteria; Actinobacteria; Streptomycetales; Streptomycetaceae; Streptomyces; Streptomyces sviceus [WP_007385613.1]
Bacteria; Actinobacteria; Streptomycetales; Streptomycetaceae; Streptomyces; Streptomyces torulosus [WP_055717500.1 WP_055712040.1]
Bacteria; Actinobacteria; Streptomycetales; Streptomycetaceae; Streptomyces; Streptomyces turgidiscabies Car8 [ELP63788.1]
Bacteria; Actinobacteria; Streptomycetales; Streptomycetaceae; Streptomyces; Streptomyces turgidiscabies [GAQ72777.1 GAQ71470.1 WP_006374944.1]
Bacteria; Actinobacteria; Streptomycetales; Streptomycetaceae; Streptomyces; Streptomyces violaceoruber [WP_030945068.1 WP_030948770.1]
Bacteria; Actinobacteria; Streptomycetales; Streptomycetaceae; Streptomyces; Streptomyces viridochromogenes Tue57 [ELS52838.1]
Bacteria; Actinobacteria; Streptomycetales; Streptomycetaceae; Streptomyces; Streptomyces viridochromogenes [WP_004003565.1 WP_004003662.1 WP_037889330.1]
Bacteria; Actinobacteria; Streptomycetales; Streptomycetaceae; Streptomyces; Streptomyces xylophagus [WP_043664400.1 WP_078651258.1]
Bacteria; Actinobacteria; Streptomycetales; Streptomycetaceae; Streptomyces; Streptomyces cinnamoneus group; Streptomyces cinnamoneus [WP_071962019.1]
Bacteria; Actinobacteria; Streptosporangiales; Thermomonosporaceae; Actinomadura oligospora [WP_051466705.1]
Bacteria; Bacteroidetes; Cytophagia; Cytophagales; Cytophagaceae; Sporocytophaga; Sporocytophaga myxococcoides [GAL83605.1 WP_045459194.1]
Bacteria; Bacteroidetes; Cytophagia; Cytophagales; Flammeovirgaceae; Fabibacter; Fabibacter misakiensis [WP_069833806.1]
Bacteria; Bacteroidetes; Cytophagia; Cytophagales; Hymenobacteraceae; Pontibacter; Pontibacter sp. S10-8 [WP_073852358.1]
Bacteria; Bacteroidetes; Sphingobacteriia; Sphingobacteriales; Sphingobacteriaceae; Sphingobacterium; Sphingobacterium lactis [SEG23904.1]
Bacteria; Deinococcus-Thermus; Deinococci; Deinococcales; Deinococcaceae; Deinococcus; Deinococcus hopiensis [WP_084045024.1]
Bacteria; Firmicutes; Bacilli; Bacillales; Listeriaceae; Listeria; Listeria fleischmannii 1991 [KMT61426.1]
Bacteria; Firmicutes; Bacilli; Bacillales; Listeriaceae; Listeria; Listeria fleischmannii [WP_077947259.1 WP_051057325.1]
Bacteria; Firmicutes; Bacilli; Bacillales; Listeriaceae; Listeria; Listeria floridensis [WP_036097424.1]
Bacteria; Firmicutes; Bacilli; Lactobacillales; Enterococcaceae; Melissococcus; Melissococcus plutonius [WP_015695480.1 WP_013774659.1 WP_048589586.1]
Bacteria; Firmicutes; Bacilli; Lactobacillales; Streptococcaceae; Streptococcus; Streptococcus oralis [KXT80651.1 WP_061415716.1]
Bacteria; Firmicutes; Bacilli; Lactobacillales; Streptococcaceae; Streptococcus; Streptococcus sp. DD10 [WP_082782057.1]
Bacteria; Firmicutes; Bacilli; Lactobacillales; Streptococcaceae; Streptococcus; Streptococcus sp. DD11 [KXT85708.1]
Bacteria; Proteobacteria; Alphaproteobacteria; Alphaproteobacteria bacterium 13_2_20CM_2_64_7 [OLB76146.1]
Bacteria; Proteobacteria; Alphaproteobacteria; Rhizobiales; Rhizobiales bacterium 62-17 [OJY03981.1]
Bacteria; Proteobacteria; Alphaproteobacteria; Rhizobiales; Beijerinckiaceae; Beijerinckia; Beijerinckia sp. 28-YEA-48 [SEB67174.1 SEB56691.1]
Bacteria; Proteobacteria; Alphaproteobacteria; Rhizobiales; Bradyrhizobiaceae; Bradyrhizobium; Bradyrhizobium sp. WSM1253 [WP_051058437.1 EIG59144.1]
Bacteria; Proteobacteria; Alphaproteobacteria; Rhizobiales; Hyphomicrobiaceae; Rhodoplanes; Rhodoplanes sp. Z2-YC6860 [WP_068021390.1 WP_068026824.1]
Bacteria; Proteobacteria; Alphaproteobacteria; Rhizobiales; Phyllobacteriaceae; Chelativorans; Chelativorans sp. BNC1 [WP_011582253.1]

Bacteria; Proteobacteria; Alphaproteobacteria; Rhizobiales; Phyllobacteriaceae; Mesorhizobium; Mesorhizobium prunaredense [WP_077373955.1]
Bacteria; Proteobacteria; Alphaproteobacteria; Rhizobiales; Phyllobacteriaceae; Mesorhizobium; Mesorhizobium sp. L103C105A0 [WP_023833407.1]
Bacteria; Proteobacteria; Alphaproteobacteria; Rhizobiales; Phyllobacteriaceae; Mesorhizobium; Mesorhizobium sp. LNJC405B00 [ESX84577.1 WP_032999681.1]
Bacteria; Proteobacteria; Alphaproteobacteria; Rhizobiales; Rhizobiaceae; Rhizobium/Agrobacterium group [WP_083531097.1]
Bacteria; Proteobacteria; Alphaproteobacteria; Rhizobiales; Rhizobiaceae; Rhizobium/Agrobacterium group; Rhizobium; Rhizobium etli [WP_086084422.1]
Bacteria; Proteobacteria; Alphaproteobacteria; Rhizobiales; Rhizobiaceae; Rhizobium/Agrobacterium group; Rhizobium; Rhizobium leguminosarum [WP_024323662.1 WP_085994747.1 WP_003577012.1]
Bacteria; Proteobacteria; Alphaproteobacteria; Rhizobiales; Rhizobiaceae; Rhizobium/Agrobacterium group; Rhizobium; Rhizobium phaseoli [WP_064824106.1]
Bacteria; Proteobacteria; Alphaproteobacteria; Rhizobiales; Rhizobiaceae; Rhizobium/Agrobacterium group; Rhizobium; Rhizobium sp. 58 [OJF98227.1]
Bacteria; Proteobacteria; Alphaproteobacteria; Rhizobiales; Rhizobiaceae; Sinorhizobium/Ensifer group; Sinorhizobium; Sinorhizobium sp. GW3 [WP_060603084.1]
Bacteria; Proteobacteria; Alphaproteobacteria; Rhodobacterales; Rhodobacteraceae; Phaeobacter; Phaeobacter inhibens [WP_014875122.1 WP_027246973.1 WP_014880185.1]
Bacteria; Proteobacteria; Alphaproteobacteria; Rhodobacterales; Rhodobacteraceae; Roseovarius; Roseovarius aestuarii [WP_085802561.1 WP_085799196.1]
Bacteria; Proteobacteria; Alphaproteobacteria; Rhodospirillales; Acetobacteraceae; Roseomonas; Roseomonas stagni DSM 19981 [SFK72557.1]
Bacteria; Proteobacteria; Betaproteobacteria; Burkholderiales; Alcaligenaceae; Achromobacter; Achromobacter piechaudii [WP_050730750.1 WP_061306320.1 KNY06773.1]
Bacteria; Proteobacteria; Betaproteobacteria; Burkholderiales; Alcaligenaceae; Achromobacter; Achromobacter xylosoxidans [WP_081259856.1 OAE56763.1]
Bacteria; Proteobacteria; Betaproteobacteria; Burkholderiales; Burkholderiaceae; Burkholderia; Burkholderia sp. Bp7605 [WP_069233796.1 AOK28758.1]
Bacteria; Proteobacteria; Betaproteobacteria; Burkholderiales; Burkholderiaceae; Burkholderia; Burkholderia sp. LMG 28154 [WP_089339826.1]
Bacteria; Proteobacteria; Betaproteobacteria; Burkholderiales; Burkholderiaceae; Burkholderia; Burkholderia cepacia complex; Burkholderia cenocepacia [WP_077217522.1]
Bacteria; Proteobacteria; Betaproteobacteria; Burkholderiales; Burkholderiaceae; Burkholderia; pseudomallei group; Burkholderia pseudomallei 576 [EEC35331.1]
Bacteria; Proteobacteria; Betaproteobacteria; Burkholderiales; Burkholderiaceae; Burkholderia; pseudomallei group; Burkholderia pseudomallei [KGD28292.1 WP_050809000.1 WP_038730963.1]
Bacteria; Proteobacteria; Betaproteobacteria; Burkholderiales; Burkholderiaceae; Burkholderia; pseudomallei group; Burkholderia sp. TSV85 [WP_059515959.1 KVE27988.1]
Bacteria; Proteobacteria; Betaproteobacteria; Burkholderiales; Burkholderiaceae; Burkholderia; pseudomallei group; Burkholderia sp. TSV86 [WP_059569078.1]
Bacteria; Proteobacteria; Betaproteobacteria; Burkholderiales; Burkholderiaceae; Lautropia; Lautropia mirabilis [WP_005674690.1]
Bacteria; Proteobacteria; Betaproteobacteria; Burkholderiales; Comamonadaceae; Acidovorax; Acidovorax avenae [WP_013595414.1 WP_053845705.1]
Bacteria; Proteobacteria; Betaproteobacteria; Burkholderiales; Comamonadaceae; Acidovorax; Acidovorax delafieldii [WP_005797707.1]
Bacteria; Proteobacteria; Betaproteobacteria; Burkholderiales; Comamonadaceae; Acidovorax; Acidovorax radicis [WP_010460852.1]
Bacteria; Proteobacteria; Betaproteobacteria; Burkholderiales; Comamonadaceae; Pelomonas; Pelomonas sp. Root1217 [WP_057296074.1]
Bacteria; Proteobacteria; Betaproteobacteria; Burkholderiales; Comamonadaceae; Pseudacidovorax; Pseudacidovorax sp. RU35E [SIP91266.1 WP_083715780.1]
Bacteria; Proteobacteria; Betaproteobacteria; Burkholderiales; Methylibium; Methylibium sp. CF059 [WP_052197976.1]

Bacteria; Proteobacteria; Betaproteobacteria; Burkholderiales; Rhizobacter; Rhizobacter sp. OV335 [WP_073466190.1]
Bacteria; Proteobacteria; Betaproteobacteria; Neisseriales; Neisseriaceae; Bergeriella; Bergeriella denitrificans [WP_066078123.1]
Bacteria; Proteobacteria; Gammaproteobacteria; Alteromonadales; Pseudoalteromonadaceae; Pseudoalteromonas; Pseudoalteromonas aliena [WP_077535483.1]
Bacteria; Proteobacteria; Gammaproteobacteria; Enterobacterales; Enterobacteriaceae; Enterobacter [WP_080562868.1]
Bacteria; Proteobacteria; Gammaproteobacteria; Enterobacterales; Enterobacteriaceae; Enterobacteriaceae bacterium B14 [WP_051014477.1]
Bacteria; Proteobacteria; Gammaproteobacteria; Enterobacterales; Enterobacteriaceae; Enterobacter; Enterobacter soli [WP_014063834.1]
Bacteria; Proteobacteria; Gammaproteobacteria; Enterobacterales; Morganellaceae; Xenorhabdus; Xenorhabdus bovienii str. kraussei Becker Underwood [CDH26862.1]
Bacteria; Proteobacteria; Gammaproteobacteria; Enterobacterales; Morganellaceae; Xenorhabdus; Xenorhabdus bovienii [WP_051875904.1]
Bacteria; Proteobacteria; Gammaproteobacteria; Enterobacterales; Pectobacteriaceae; Dickeya [WP_035346410.1 WP_038919072.1 WP_038658440.1 WP_035342496.1]
Bacteria; Proteobacteria; Gammaproteobacteria; Enterobacterales; Pectobacteriaceae; Dickeya; Dickeya chrysanthemi [WP_033576735.1 WP_038658642.1 WP_012769725.1 WP_040001065.1]
Bacteria; Proteobacteria; Gammaproteobacteria; Enterobacterales; Pectobacteriaceae; Dickeya; Dickeya dadantii 3937 [ADM98571.1]
Bacteria; Proteobacteria; Gammaproteobacteria; Enterobacterales; Pectobacteriaceae; Dickeya; Dickeya dadantii [WP_077245618.1 WP_038901269.1 WP_033111867.1 WP_013317947.1 WP_038922882.1 WP_077245097.1 WP_081642641.1 WP_081641813.1 WP_050570147.1]
Bacteria; Proteobacteria; Gammaproteobacteria; Enterobacterales; Pectobacteriaceae; Dickeya; Dickeya dianthicola [WP_024106007.1]
Bacteria; Proteobacteria; Gammaproteobacteria; Enterobacterales; Pectobacteriaceae; Dickeya; Dickeya solani D s0432-1 [ERO58952.1]
Bacteria; Proteobacteria; Gammaproteobacteria; Enterobacterales; Pectobacteriaceae; Dickeya; Dickeya solani [WP_039694696.1 WP_039695566.1 WP_057085538.1 WP_022633652.1 WP_087881289.1 WP_051120789.1 WP_057083442.1]
Bacteria; Proteobacteria; Gammaproteobacteria; Enterobacterales; Pectobacteriaceae; Dickeya; Dickeya sp. 2B12 [WP_033568797.1 WP_033568852.1]
Bacteria; Proteobacteria; Gammaproteobacteria; Enterobacterales; Pectobacteriaceae; Dickeya; Dickeya sp. NCPPB 3274 [WP_042859844.1 WP_042859903.1]
Bacteria; Proteobacteria; Gammaproteobacteria; Enterobacterales; Pectobacteriaceae; Dickeya; Dickeya sp. S1 [WP_049854002.1 WP_049853692.1]
Bacteria; Proteobacteria; Gammaproteobacteria; Enterobacterales; Pectobacteriaceae; Dickeya; Dickeya zeae [WP_012886601.1 WP_019845396.1 WP_038925860.1 WP_038904286.1 WP_038916143.1 WP_019845586.1 WP_023639620.1 WP_016941422.1 WP_012884670.1 WP_038907878.1]
Bacteria; Proteobacteria; Gammaproteobacteria; Enterobacterales; Pectobacteriaceae; Pectobacterium; Pectobacterium atrosepticum [WP_039291927.1 WP_011093695.1]
Bacteria; Proteobacteria; Gammaproteobacteria; Enterobacterales; Pectobacteriaceae; Pectobacterium; Pectobacterium betavasculorum [WP_039299648.1 WP_039322906.1]
Bacteria; Proteobacteria; Gammaproteobacteria; Enterobacterales; Pectobacteriaceae; Pectobacterium; Pectobacterium carotovorum subsp. odoriferum [AIU88614.1]
Bacteria; Proteobacteria; Gammaproteobacteria; Enterobacterales; Pectobacteriaceae; Pectobacterium; Pectobacterium carotovorum [KHN50660.1 WP_052198383.1 WP_039345091.1 WP_015840400.1 WP_052903354.1 WP_039496408.1 WP_039544759.1 WP_039468236.1 WP_039312045.1 WP_052900783.1 WP_014915523.1 WP_039518308.1 WP_010295561.1 WP_039489348.1 WP_039279715.1 WP_015839605.1 WP_048259997.1 WP_039314807.1 WP_010285654.1 WP_039537654.1 WP_039505673.1 WP_039473296.1 WP_039356773.1 WP_039558453.1 WP_039551019.1 WP_039487678.1 WP_039286330.1 WP_014914751.1 WP_010281412.1 WP_039358884.1 WP_039509305.1 WP_044203322.1 WP_048259399.1 WP_039280078.1 WP_010296828.1]
Bacteria; Proteobacteria; Gammaproteobacteria; Enterobacterales; Pectobacteriaceae; Pectobacterium; Pectobacterium parmentieri WPP163 [ACX88752.1]
Bacteria; Proteobacteria; Gammaproteobacteria; Enterobacterales; Pectobacteriaceae; Pectobacterium; Pectobacterium parmentieri [WP_043988974.1 WP_025918673.1 WP_015730584.1 WP_033071752.1]

Bacteria; Proteobacteria; Gammaproteobacteria; Enterobacterales; Pectobacteriaceae; Pectobacterium; Pectobacterium sp. SCC3193
[AFI91073.1 WP_043899092.1 WP_014700146.1]
Bacteria; Proteobacteria; Gammaproteobacteria; Enterobacterales; Pectobacteriaceae; Pectobacterium; Pectobacterium wasabiae
[WP_005974214.1 WP_005974116.1]
Bacteria; Proteobacteria; Gammaproteobacteria; Enterobacterales; Yersiniaceae; Yersinia; Yersinia mollaretii ATCC 43969 [EEQ11827.1
]
Bacteria; Proteobacteria; Gammaproteobacteria; Enterobacterales; Yersiniaceae; Yersinia; Yersinia mollaretii [WP_049678750.1
WP_050413172.1 WP_050538947.1 WP_049647759.1]
Bacteria; Proteobacteria; Gammaproteobacteria; Pseudomonadales; Pseudomonadaceae; Pseudomonas [WP_031754313.1 WP_049261755.1
WP_004343737.1 WP_003122690.1 WP_016852872.1 WP_031631984.1 WP_033946109.1 WP_043555118.1 WP_025324949.1 WP_031690645.1
WP_003139302.1 WP_031653392.1 WP_030048804.1 WP_017002398.1 WP_016561915.1 WP_014602898.1 WP_031634552.1 WP_033970721.1
WP_031628248.1 WP_025921232.1]
Bacteria; Proteobacteria; Gammaproteobacteria; Pseudomonadales; Pseudomonadaceae; Pseudomonas; Pseudomonas aeruginosa 3575
[EZO20093.1]
Bacteria; Proteobacteria; Gammaproteobacteria; Pseudomonadales; Pseudomonadaceae; Pseudomonas; Pseudomonas aeruginosa 62
[ERX80597.1]
Bacteria; Proteobacteria; Gammaproteobacteria; Pseudomonadales; Pseudomonadaceae; Pseudomonas; Pseudomonas aeruginosa BL01
[ERY65812.1]
Bacteria; Proteobacteria; Gammaproteobacteria; Pseudomonadales; Pseudomonadaceae; Pseudomonas; Pseudomonas aeruginosa BL04
[ERV68420.1]
Bacteria; Proteobacteria; Gammaproteobacteria; Pseudomonadales; Pseudomonadaceae; Pseudomonas; Pseudomonas aeruginosa BL07
[ERV73748.1]
Bacteria; Proteobacteria; Gammaproteobacteria; Pseudomonadales; Pseudomonadaceae; Pseudomonas; Pseudomonas aeruginosa BL09
[ERV62887.1]
Bacteria; Proteobacteria; Gammaproteobacteria; Pseudomonadales; Pseudomonadaceae; Pseudomonas; Pseudomonas aeruginosa BL13
[ERY33074.1]
Bacteria; Proteobacteria; Gammaproteobacteria; Pseudomonadales; Pseudomonadaceae; Pseudomonas; Pseudomonas aeruginosa BL15
[ERV32818.1]
Bacteria; Proteobacteria; Gammaproteobacteria; Pseudomonadales; Pseudomonadaceae; Pseudomonas; Pseudomonas aeruginosa BL19
[ERV15545.1]
Bacteria; Proteobacteria; Gammaproteobacteria; Pseudomonadales; Pseudomonadaceae; Pseudomonas; Pseudomonas aeruginosa BWH030
[EZN70173.1]
Bacteria; Proteobacteria; Gammaproteobacteria; Pseudomonadales; Pseudomonadaceae; Pseudomonas; Pseudomonas aeruginosa BWH054
[EZP02724.1]
Bacteria; Proteobacteria; Gammaproteobacteria; Pseudomonadales; Pseudomonadaceae; Pseudomonas; Pseudomonas aeruginosa BWH055
[EZO83679.1]
Bacteria; Proteobacteria; Gammaproteobacteria; Pseudomonadales; Pseudomonadaceae; Pseudomonas; Pseudomonas aeruginosa BWH057
[EZO59250.1]
Bacteria; Proteobacteria; Gammaproteobacteria; Pseudomonadales; Pseudomonadaceae; Pseudomonas; Pseudomonas aeruginosa BWHPSA020
[ERW37676.1]
Bacteria; Proteobacteria; Gammaproteobacteria; Pseudomonadales; Pseudomonadaceae; Pseudomonas; Pseudomonas aeruginosa BWHPSA021
[ERW22682.1]
Bacteria; Proteobacteria; Gammaproteobacteria; Pseudomonadales; Pseudomonadaceae; Pseudomonas; Pseudomonas aeruginosa BWHPSA026
[ERV84541.1]
Bacteria; Proteobacteria; Gammaproteobacteria; Pseudomonadales; Pseudomonadaceae; Pseudomonas; Pseudomonas aeruginosa BWHPSA041
[ETV39073.1]
Bacteria; Proteobacteria; Gammaproteobacteria; Pseudomonadales; Pseudomonadaceae; Pseudomonas; Pseudomonas aeruginosa BWHPSA046
[ETV08427.1]
Bacteria; Proteobacteria; Gammaproteobacteria; Pseudomonadales; Pseudomonadaceae; Pseudomonas; Pseudomonas aeruginosa BWHPSA047
[ETV04207.1]

Bacteria; Proteobacteria; Gammaproteobacteria; Pseudomonadales; Pseudomonadaceae; Pseudomonas; Pseudomonas aeruginosa C41
[ERU67372.1]
Bacteria; Proteobacteria; Gammaproteobacteria; Pseudomonadales; Pseudomonadaceae; Pseudomonas; Pseudomonas aeruginosa CF18
[ERX57339.1]
Bacteria; Proteobacteria; Gammaproteobacteria; Pseudomonadales; Pseudomonadaceae; Pseudomonas; Pseudomonas aeruginosa CF5
[ERZ27429.1]
Bacteria; Proteobacteria; Gammaproteobacteria; Pseudomonadales; Pseudomonadaceae; Pseudomonas; Pseudomonas aeruginosa IGB83
[KAJ07225.1]
Bacteria; Proteobacteria; Gammaproteobacteria; Pseudomonadales; Pseudomonadaceae; Pseudomonas; Pseudomonas aeruginosa LES431
[AHC65572.1]
Bacteria; Proteobacteria; Gammaproteobacteria; Pseudomonadales; Pseudomonadaceae; Pseudomonas; Pseudomonas aeruginosa M10
[KAJ16447.1]
Bacteria; Proteobacteria; Gammaproteobacteria; Pseudomonadales; Pseudomonadaceae; Pseudomonas; Pseudomonas aeruginosa M8A.2
[ERU97551.1]
Bacteria; Proteobacteria; Gammaproteobacteria; Pseudomonadales; Pseudomonadaceae; Pseudomonas; Pseudomonas aeruginosa M8A.4
[ERX88843.1]
Bacteria; Proteobacteria; Gammaproteobacteria; Pseudomonadales; Pseudomonadaceae; Pseudomonas; Pseudomonas aeruginosa MH27
[CDH77232.1]
Bacteria; Proteobacteria; Gammaproteobacteria; Pseudomonadales; Pseudomonadaceae; Pseudomonas; Pseudomonas aeruginosa MH38
[CDH71700.1]
Bacteria; Proteobacteria; Gammaproteobacteria; Pseudomonadales; Pseudomonadaceae; Pseudomonas; Pseudomonas aeruginosa MPAO1/P1
[EHS34367.1]
Bacteria; Proteobacteria; Gammaproteobacteria; Pseudomonadales; Pseudomonadaceae; Pseudomonas; Pseudomonas aeruginosa MPAO1/P2
[EHS36088.1]
Bacteria; Proteobacteria; Gammaproteobacteria; Pseudomonadales; Pseudomonadaceae; Pseudomonas; Pseudomonas aeruginosa MTB-1
[AHB55897.1]
Bacteria; Proteobacteria; Gammaproteobacteria; Pseudomonadales; Pseudomonadaceae; Pseudomonas; Pseudomonas aeruginosa NCMG1179
[GAA17963.1]
Bacteria; Proteobacteria; Gammaproteobacteria; Pseudomonadales; Pseudomonadaceae; Pseudomonas; Pseudomonas aeruginosa P47
[OPF27848.1]
Bacteria; Proteobacteria; Gammaproteobacteria; Pseudomonadales; Pseudomonadaceae; Pseudomonas; Pseudomonas aeruginosa PA1R
[AHA21955.1]
Bacteria; Proteobacteria; Gammaproteobacteria; Pseudomonadales; Pseudomonadaceae; Pseudomonas; Pseudomonas aeruginosa PA21_ST175
[EME91390.1]
Bacteria; Proteobacteria; Gammaproteobacteria; Pseudomonadales; Pseudomonadaceae; Pseudomonas; Pseudomonas aeruginosa PA38182
[CDI90661.1]
Bacteria; Proteobacteria; Gammaproteobacteria; Pseudomonadales; Pseudomonadaceae; Pseudomonas; Pseudomonas aeruginosa PA7
[ABR85278.1]
Bacteria; Proteobacteria; Gammaproteobacteria; Pseudomonadales; Pseudomonadaceae; Pseudomonas; Pseudomonas aeruginosa PAK
[EOT15987.1]
Bacteria; Proteobacteria; Gammaproteobacteria; Pseudomonadales; Pseudomonadaceae; Pseudomonas; Pseudomonas aeruginosa PS75
[EZO52610.1]
Bacteria; Proteobacteria; Gammaproteobacteria; Pseudomonadales; Pseudomonadaceae; Pseudomonas; Pseudomonas aeruginosa RB
[GAJ56493.1]
Bacteria; Proteobacteria; Gammaproteobacteria; Pseudomonadales; Pseudomonadaceae; Pseudomonas; Pseudomonas aeruginosa UDL
[ERX59788.1]
Bacteria; Proteobacteria; Gammaproteobacteria; Pseudomonadales; Pseudomonadaceae; Pseudomonas; Pseudomonas aeruginosa VRFPA08
[ETD41895.1]
Bacteria; Proteobacteria; Gammaproteobacteria; Pseudomonadales; Pseudomonadaceae; Pseudomonas; Pseudomonas aeruginosa VRFPA09
[EVT85841.1]

Bacteria; Proteobacteria; Gammaproteobacteria; Pseudomonadales; Pseudomonadaceae; Pseudomonas; Pseudomonas aeruginosa WS136 [CDM45833.1]
Bacteria; Proteobacteria; Gammaproteobacteria; Pseudomonadales; Pseudomonadaceae; Pseudomonas; Pseudomonas aeruginosa X13273 [ERX21417.1]
Bacteria; Proteobacteria; Gammaproteobacteria; Pseudomonadales; Pseudomonadaceae; Pseudomonas; Pseudomonas aeruginosa X24509 [ERX59836.1]
Bacteria; Proteobacteria; Gammaproteobacteria; Pseudomonadales; Pseudomonadaceae; Pseudomonas; Pseudomonas aeruginosa c7447m [AGV66704.1]
Bacteria; Proteobacteria; Gammaproteobacteria; Pseudomonadales; Pseudomonadaceae; Pseudomonas; Pseudomonas aeruginosa str. Stone 130 [EMZ59143.1]
Bacteria; Proteobacteria; Gammaproteobacteria; Pseudomonadales; Pseudomonadaceae; Pseudomonas; Pseudomonas aeruginosa [WP_065429384.1 WP_078456940.1 WP_034025894.1 WP_034006371.1 WP_060961411.1 KYO99703.1 WP_031674067.1 KYO85056.1 WP_031635467.1 WP_043096130.1 WP_031758954.1 WP_031675662.1 WP_023082412.1 WP_034059890.1 WP_041127196.1 WP_031760648.1 WP_031754878.1 WP_074244339.1 WP_033988912.1 WP_033940762.1 WP_087935274.1 WP_073654609.1 WP_034003781.1 WP_073659871.1 WP_034044990.1 CEI21178.1 WP_033993592.1 WP_033876238.1 WP_083567670.1 WP_058162405.1 CKH43460.1 WP_031757861.1 WP_034076059.1 WP_048521089.1 WP_031684590.1 WP_079452834.1 WP_060627976.1 WP_012614143.1 WP_034033931.1 WP_033974538.1 WP_058200184.1 WP_058139672.1 WP_086339893.1 WP_034011581.1 WP_031642714.1 WP_031761345.1 WP_003089517.1 WP_031692924.1 WP_034004207.1 WP_034074852.1 WP_044061518.1 WP_033997622.1 WP_031636393.1 WP_031642557.1 WP_033995126.1 WP_041025347.1 WP_088145242.1 WP_031637106.1 WP_031691683.1 WP_079200326.1 WP_043545733.1 WP_033983977.1 WP_031653214.1 WP_049237253.1 WP_033954720.1 WP_070341648.1 WP_031639881.1 WP_025271100.1 WP_033982205.1 WP_033938604.1 WP_087823971.1 WP_058354535.1 WP_015502885.1 WP_034048336.1 WP_049302056.1 WP_034084564.1 WP_031634398.1 WP_031633128.1 WP_048303748.1 WP_031629435.1 WP_031629014.1 WP_019486286.1 WP_049951040.1 WP_042179036.1 WP_046895974.1 WP_023434389.1 WP_058134151.1 WP_031676048.1 WP_074222397.1 WP_070338465.1 WP_034053070.1 WP_031641066.1 KYO75305.1 WP_019726219.1 WP_058148778.1 WP_057380221.1 WP_034021772.1 WP_086250634.1 WP_033949210.1 WP_058176394.1 WP_058180201.1 WP_062842710.1 WP_031755934.1 WP_034043318.1 WP_033863101.1 WP_070344629.1 WP_058151060.1 WP_061198078.1 WP_058156197.1 WP_070345048.1 WP_073657430.1 WP_073699833.1 WP_074207057.1 WP_034014193.1 WP_021264816.1 WP_031629098.1 WP_025982335.1 WP_070330227.1 WP_057393257.1 WP_034016729.1 WP_022580992.1 WP_039028213.1 WP_031637806.1 WP_073646980.1 WP_034012827.1 WP_003124573.1 WP_033958252.1 WP_061198723.1 WP_043105079.1 WP_034000754.1 WP_033980273.1 WP_058012804.1 WP_041025624.1 WP_033996203.1 SKC13639.1 WP_073665658.1 WP_079732721.1 BAQ39857.1 WP_033893053.1 WP_033990498.1 WP_061199502.1 WP_087786013.1 WP_049950667.1 WP_050480309.1 WP_050397360.1 WP_050394893.1 CRQ68682.1 CRN99737.1 WP_050467544.1 WP_050397981.1 WP_050395105.1 WP_050395839.1 WP_050396979.1 WP_003131529.1 WP_049290563.1 WP_079459273.1 OES65317.1]
Bacteria; Proteobacteria; Gammaproteobacteria; Pseudomonadales; Pseudomonadaceae; Pseudomonas; Pseudomonas denitrificans (nomen rejiciendum) [WP_049321509.1]
Bacteria; Proteobacteria; Gammaproteobacteria; Pseudomonadales; Pseudomonadaceae; Pseudomonas; Pseudomonas sp. 2_1_26 [EHF09421.1 WP_031627764.1]
Bacteria; Proteobacteria; Gammaproteobacteria; Pseudomonadales; Pseudomonadaceae; Pseudomonas; Pseudomonas sp. NFACC56-3 [SCW36062.1]
Bacteria; Proteobacteria; Gammaproteobacteria; Pseudomonadales; Pseudomonadaceae; Pseudomonas; Pseudomonas sp. P179 [EMZ59426.1]
Bacteria; Proteobacteria; Gammaproteobacteria; Thiotrichales; Thiotrichaceae; Thiothrix; Thiothrix flexilis [WP_020560061.1]
Bacteria; Proteobacteria; Gammaproteobacteria; Vibrionales; Vibrionaceae; Vibrio; Vibrio aerogenes [WP_073603194.1 WP_073605707.1]
Bacteria; Proteobacteria; Gammaproteobacteria; Vibrionales; Vibrionaceae; Vibrio; Vibrio mangrovi [WP_087480881.1]
Bacteria; Proteobacteria; Gammaproteobacteria; Vibrionales; Vibrionaceae; Vibrio; Vibrio quintilis [WP_073581450.1]
Bacteria; Proteobacteria; Gammaproteobacteria; Xanthomonadales; Xanthomonadaceae; Lysobacter; Lysobacter antibioticus [WP_057916419.1 WP_057972939.1 ALN78647.1 ALN65933.1]
Bacteria; Proteobacteria; Gammaproteobacteria; Xanthomonadales; Xanthomonadaceae; Stenotrophomonas; Stenotrophomonas chelatiphaga [WP_057508446.1]
Eukaryota; Fungi; fungal sp. No.14919 [GAW22126.1 GAW17550.1]
Eukaryota; Fungi; Dikarya; Ascomycota; Pezizomycotina; Dothideomycetes; Dothideomycetes incertae sedis; Botryosphaeriales; Botryosphaeriaceae; Diplodia corticola [XP_020125814.1]
Eukaryota; Fungi; Dikarya; Ascomycota; Pezizomycotina; Dothideomycetes; Dothideomycetes incertae sedis; Botryosphaeriales; Botryosphaeriaceae; Diplodia seriata [OMP84235.1 OMP86698.1 KKY21108.1]

Eukaryota; Fungi; Dikarya; Ascomycota; Pezizomycotina; Dothideomycetes; Dothideomycetes incertae sedis; Botryosphaeriales; Botryosphaeriaceae; Macrophomina phaseolina MS6 [EKG19700.1]
Eukaryota; Fungi; Dikarya; Ascomycota; Pezizomycotina; Dothideomycetes; Dothideomycetes incertae sedis; Botryosphaeriales; Botryosphaeriaceae; Neofusicoccum parvum UCRNP2 [XP_007589582.1 XP_007587063.1]
Eukaryota; Fungi; Dikarya; Ascomycota; Pezizomycotina; Dothideomycetes; Dothideomycetidae; Capnodiales; Mycosphaerellaceae; Pseudocercospora fijiensis CIRAD86 [XP_007924954.1]
Eukaryota; Fungi; Dikarya; Ascomycota; Pezizomycotina; Dothideomycetes; Dothideomycetidae; Capnodiales; Mycosphaerellaceae; Sphaerulina musiva SO2202 [XP_016764308.1]
Eukaryota; Fungi; Dikarya; Ascomycota; Pezizomycotina; Dothideomycetes; Dothideomycetidae; Dothideales; Saccotheciaceae; Aureobasidium pullulans EXF-150 [KEQ79523.1]
Eukaryota; Fungi; Dikarya; Ascomycota; Pezizomycotina; Dothideomycetes; Pleosporomycetidae; Pleosporales; Massarineae; Didymosphaeriaceae; Paraphaeosphaeria sporulosa [XP_018032083.1]
Eukaryota; Fungi; Dikarya; Ascomycota; Pezizomycotina; Dothideomycetes; Pleosporomycetidae; Pleosporales; Pleosporineae; Cucurbitariaceae; Pyrenochaeta sp. DS3sAY3a [OAL49315.1]
Eukaryota; Fungi; Dikarya; Ascomycota; Pezizomycotina; Dothideomycetes; Pleosporomycetidae; Pleosporales; Pleosporineae; Didymellaceae; Ascochyta rabiei [KZM28291.1]
Eukaryota; Fungi; Dikarya; Ascomycota; Pezizomycotina; Dothideomycetes; Pleosporomycetidae; Pleosporales; Pleosporineae; Didymellaceae; Epicoccum nigrum [OSS45479.1]
Eukaryota; Fungi; Dikarya; Ascomycota; Pezizomycotina; Dothideomycetes; Pleosporomycetidae; Pleosporales; Pleosporineae; Phaeosphaeriaceae; Parastagonospora nodorum SN15 [XP_001805565.1 XP_001798237.1]
Eukaryota; Fungi; Dikarya; Ascomycota; Pezizomycotina; Dothideomycetes; Pleosporomycetidae; Pleosporales; Pleosporineae; Pleosporaceae; Alternaria; Alternaria alternata [XP_018388203.1]
Eukaryota; Fungi; Dikarya; Ascomycota; Pezizomycotina; Dothideomycetes; Pleosporomycetidae; Pleosporales; Pleosporineae; Pleosporaceae; Bipolaris maydis ATCC 48331 [XP_014074331.1 XP_014080000.1]
Eukaryota; Fungi; Dikarya; Ascomycota; Pezizomycotina; Dothideomycetes; Pleosporomycetidae; Pleosporales; Pleosporineae; Pleosporaceae; Bipolaris oryzae ATCC 44560 [XP_007688271.1 XP_007693023.1]
Eukaryota; Fungi; Dikarya; Ascomycota; Pezizomycotina; Dothideomycetes; Pleosporomycetidae; Pleosporales; Pleosporineae; Pleosporaceae; Bipolaris sorokiniana ND90Pr [XP_007704220.1 XP_007702245.1]
Eukaryota; Fungi; Dikarya; Ascomycota; Pezizomycotina; Dothideomycetes; Pleosporomycetidae; Pleosporales; Pleosporineae; Pleosporaceae; Bipolaris victoriae FI3 [XP_014555246.1]
Eukaryota; Fungi; Dikarya; Ascomycota; Pezizomycotina; Dothideomycetes; Pleosporomycetidae; Pleosporales; Pleosporineae; Pleosporaceae; Bipolaris zeicola 26-R-13 [XP_007718543.1 XP_007712846.1]
Eukaryota; Fungi; Dikarya; Ascomycota; Pezizomycotina; Dothideomycetes; Pleosporomycetidae; Pleosporales; Pleosporineae; Pleosporaceae; Pyrenophora teres f. teres 0-1 [XP_003296088.1]
Eukaryota; Fungi; Dikarya; Ascomycota; Pezizomycotina; Dothideomycetes; Pleosporomycetidae; Pleosporales; Pleosporineae; Pleosporaceae; Pyrenophora tritici-repentis Pt-1C-BFP [XP_001935579.1]
Eukaryota; Fungi; Dikarya; Ascomycota; Pezizomycotina; Dothideomycetes; Pleosporomycetidae; Pleosporales; Pleosporineae; Pleosporaceae; Setosphaeria turcica Et28A [XP_008020412.1]
Eukaryota; Fungi; Dikarya; Ascomycota; Pezizomycotina; Dothideomycetes; Pleosporomycetidae; Pleosporales; Pleosporineae; Pleosporaceae; Stemphylium lycopersici [KNG48712.1]
Eukaryota; Fungi; Dikarya; Ascomycota; Pezizomycotina; Eurotiomycetes; Chaetothyriomycetidae; Chaetothyriales; Cyphellophoraceae; Cyphellophora europaea CBS 101466 [XP_008716383.1]
Eukaryota; Fungi; Dikarya; Ascomycota; Pezizomycotina; Eurotiomycetes; Chaetothyriomycetidae; Chaetothyriales; Herpotrichiellaceae; Cladophialophora carrionii CBS 160.54 [XP_008726314.1]
Eukaryota; Fungi; Dikarya; Ascomycota; Pezizomycotina; Eurotiomycetes; Chaetothyriomycetidae; Chaetothyriales; Herpotrichiellaceae; Exophiala spinifera [XP_016238272.1]
Eukaryota; Fungi; Dikarya; Ascomycota; Pezizomycotina; Eurotiomycetes; Chaetothyriomycetidae; Chaetothyriales; Herpotrichiellaceae; Phialophora americana [KIW63795.1]
Eukaryota; Fungi; Dikarya; Ascomycota; Pezizomycotina; Eurotiomycetes; Eurotiomycetidae; Eurotiales; Aspergillaceae; Aspergillus bombycis [OGM49270.1]

Eukaryota; Fungi; Dikarya; Ascomycota; Pezizomycotina; Eurotiomycetes; Eurotiomycetidae; Eurotiales; Aspergillaceae; Aspergillus flavus AF70 [KOC09207.1]
Eukaryota; Fungi; Dikarya; Ascomycota; Pezizomycotina; Eurotiomycetes; Eurotiomycetidae; Eurotiales; Aspergillaceae; Aspergillus flavus NRRL3357 [XP_002376865.1]
Eukaryota; Fungi; Dikarya; Ascomycota; Pezizomycotina; Eurotiomycetes; Eurotiomycetidae; Eurotiales; Aspergillaceae; Aspergillus nomius NRRL 13137 [XP_015409438.1]
Eukaryota; Fungi; Dikarya; Ascomycota; Pezizomycotina; Eurotiomycetes; Eurotiomycetidae; Eurotiales; Aspergillaceae; Aspergillus oryzae [OOO14150.1]
Eukaryota; Fungi; Dikarya; Ascomycota; Pezizomycotina; Eurotiomycetes; Eurotiomycetidae; Eurotiales; Aspergillaceae; Aspergillus oryzae RIB40 [XP_001821115.2 BAE59113.1]
Eukaryota; Fungi; Dikarya; Ascomycota; Pezizomycotina; Eurotiomycetes; Eurotiomycetidae; Eurotiales; Aspergillaceae; Penicillium arizonense [OGE49340.1]
Eukaryota; Fungi; Dikarya; Ascomycota; Pezizomycotina; Eurotiomycetes; Eurotiomycetidae; Eurotiales; Aspergillaceae; Penicillium brasilianum [CEJ55366.1 OOQ88317.1]
Eukaryota; Fungi; Dikarya; Ascomycota; Pezizomycotina; Eurotiomycetes; Eurotiomycetidae; Eurotiales; Aspergillaceae; Penicillium camemberti [CRL29597.1]
Eukaryota; Fungi; Dikarya; Ascomycota; Pezizomycotina; Eurotiomycetes; Eurotiomycetidae; Eurotiales; Aspergillaceae; Penicillium decumbens [OQD66296.1]
Eukaryota; Fungi; Dikarya; Ascomycota; Pezizomycotina; Eurotiomycetes; Eurotiomycetidae; Eurotiales; Aspergillaceae; Penicillium digitatum Pd1 [XP_014532962.1]
Eukaryota; Fungi; Dikarya; Ascomycota; Pezizomycotina; Eurotiomycetes; Eurotiomycetidae; Eurotiales; Aspergillaceae; Penicillium expansum [XP_016594909.1 KGO62519.1]
Eukaryota; Fungi; Dikarya; Ascomycota; Pezizomycotina; Eurotiomycetes; Eurotiomycetidae; Eurotiales; Aspergillaceae; Penicillium nalgiovense [OQE87950.1]
Eukaryota; Fungi; Dikarya; Ascomycota; Pezizomycotina; Eurotiomycetes; Eurotiomycetidae; Eurotiales; Aspergillaceae; Penicillium polonicum [OQD60072.1]
Eukaryota; Fungi; Dikarya; Ascomycota; Pezizomycotina; Eurotiomycetes; Eurotiomycetidae; Eurotiales; Aspergillaceae; Penicillium solitum [OQD93935.1]
Eukaryota; Fungi; Dikarya; Ascomycota; Pezizomycotina; Eurotiomycetes; Eurotiomycetidae; Eurotiales; Aspergillaceae; Penicillium steckii [OQE31778.1]
Eukaryota; Fungi; Dikarya; Ascomycota; Pezizomycotina; Eurotiomycetes; Eurotiomycetidae; Eurotiales; Aspergillaceae; Penicillium subrubescens [OKO99251.1]
Eukaryota; Fungi; Dikarya; Ascomycota; Pezizomycotina; Eurotiomycetes; Eurotiomycetidae; Eurotiales; Trichocomaceae; Talaromyces cellulolyticus [GAM37456.1]
Eukaryota; Fungi; Dikarya; Ascomycota; Pezizomycotina; Eurotiomycetes; Eurotiomycetidae; Eurotiales; Trichocomaceae; Talaromyces verruculosus [KUL86981.1]
Eukaryota; Fungi; Dikarya; Ascomycota; Pezizomycotina; Eurotiomycetes; Eurotiomycetidae; Onygenales; Ascosphaeraceae; Ascosphaera apis ARSEF 7405 [KZZ89047.1]
Eukaryota; Fungi; Dikarya; Ascomycota; Pezizomycotina; Leotiomycetes; Helotiales; Dermateaceae; Marssonina brunnea f. sp. 'multigermtubi' MB_m1 [XP_007297585.1]
Eukaryota; Fungi; Dikarya; Ascomycota; Pezizomycotina; Leotiomycetes; Helotiales; Helotiales incertae sedis; Phialocephala; Phialocephala subalpina [CZR55928.1]
Eukaryota; Fungi; Dikarya; Ascomycota; Pezizomycotina; Leotiomycetes; Helotiales; Helotiales incertae sedis; Rhynchosporium agropyri [CZT10615.1]
Eukaryota; Fungi; Dikarya; Ascomycota; Pezizomycotina; Leotiomycetes; Helotiales; Helotiales incertae sedis; Rhynchosporium commune [CZT10694.1]
Eukaryota; Fungi; Dikarya; Ascomycota; Pezizomycotina; Leotiomycetes; Helotiales; Helotiales incertae sedis; Rhynchosporium secalis [CZT53465.1]
Eukaryota; Fungi; Dikarya; Ascomycota; Pezizomycotina; Leotiomycetes; Helotiales; Sclerotiniaceae; Botrytis cinerea B05.10 [XP_001552651.1]

Eukaryota; Fungi; Dikarya; Ascomycota; Pezizomycotina; Leotiomycetes; Helotiales; Sclerotiniaceae; Botrytis cinerea BcDW1 [EMR88365.1]
Eukaryota; Fungi; Dikarya; Ascomycota; Pezizomycotina; Leotiomycetes; Helotiales; Sclerotiniaceae; Botrytis cinerea T4 [CCD34676.1]
Eukaryota; Fungi; Dikarya; Ascomycota; Pezizomycotina; Leotiomycetes; Helotiales; Sclerotiniaceae; Sclerotinia borealis F-4128 [ESZ90677.1]
Eukaryota; Fungi; Dikarya; Ascomycota; Pezizomycotina; Leotiomycetes; Helotiales; Sclerotiniaceae; Sclerotinia sclerotiorum 1980 UF-70 [XP_001593454.1]
Eukaryota; Fungi; Dikarya; Ascomycota; Pezizomycotina; Leotiomycetes; Leotiomycetes incertae sedis; Pseudeurotiaceae; Pseudogymnoascus sp. VKM F-4520 (FW-2644) [KFZ03420.1]
Eukaryota; Fungi; Dikarya; Ascomycota; Pezizomycotina; Sordariomycetes; Hypocreomycetidae; Glomerellales; Glomerellaceae; Colletotrichum chlorophyti [OLN85457.1 OLN97823.1]
Eukaryota; Fungi; Dikarya; Ascomycota; Pezizomycotina; Sordariomycetes; Hypocreomycetidae; Glomerellales; Glomerellaceae; Colletotrichum fioriniae PJ7 [XP_007596234.1 XP_007595117.1 XP_007595595.1 XP_007591324.1]
Eukaryota; Fungi; Dikarya; Ascomycota; Pezizomycotina; Sordariomycetes; Hypocreomycetidae; Glomerellales; Glomerellaceae; Colletotrichum gloeosporioides Cg-14 [EQB47635.1 EQB52928.1 EQB58674.1]
Eukaryota; Fungi; Dikarya; Ascomycota; Pezizomycotina; Sordariomycetes; Hypocreomycetidae; Glomerellales; Glomerellaceae; Colletotrichum gloeosporioides Nara gc5 [XP_007275031.1 XP_007286342.1 XP_007281238.1]
Eukaryota; Fungi; Dikarya; Ascomycota; Pezizomycotina; Sordariomycetes; Hypocreomycetidae; Glomerellales; Glomerellaceae; Colletotrichum graminicola M1.001 [XP_008099895.1]
Eukaryota; Fungi; Dikarya; Ascomycota; Pezizomycotina; Sordariomycetes; Hypocreomycetidae; Glomerellales; Glomerellaceae; Colletotrichum higginsianum IMI 349063 [XP_018161719.1 XP_018159399.1]
Eukaryota; Fungi; Dikarya; Ascomycota; Pezizomycotina; Sordariomycetes; Hypocreomycetidae; Glomerellales; Glomerellaceae; Colletotrichum higginsianum [CCF41121.1 CCF41727.1]
Eukaryota; Fungi; Dikarya; Ascomycota; Pezizomycotina; Sordariomycetes; Hypocreomycetidae; Glomerellales; Glomerellaceae; Colletotrichum incanum [KZL82660.1]
Eukaryota; Fungi; Dikarya; Ascomycota; Pezizomycotina; Sordariomycetes; Hypocreomycetidae; Glomerellales; Glomerellaceae; Colletotrichum nymphaeae SA-01 [KXH64666.1 KXH29882.1 KXH48297.1 KXH46358.1]
Eukaryota; Fungi; Dikarya; Ascomycota; Pezizomycotina; Sordariomycetes; Hypocreomycetidae; Glomerellales; Glomerellaceae; Colletotrichum orbiculare MAFF 240422 [ENH88181.1 ENH85153.1]
Eukaryota; Fungi; Dikarya; Ascomycota; Pezizomycotina; Sordariomycetes; Hypocreomycetidae; Glomerellales; Glomerellaceae; Colletotrichum orchidophilum [OHF02677.1 OHE92135.1 OHE91299.1]
Eukaryota; Fungi; Dikarya; Ascomycota; Pezizomycotina; Sordariomycetes; Hypocreomycetidae; Glomerellales; Glomerellaceae; Colletotrichum salicis [KXH67416.1 KXH39530.1 KXH68101.1]
Eukaryota; Fungi; Dikarya; Ascomycota; Pezizomycotina; Sordariomycetes; Hypocreomycetidae; Glomerellales; Glomerellaceae; Colletotrichum simmondsii [KXH30840.1 KXH25201.1 KXH47419.1 KXH42732.1]
Eukaryota; Fungi; Dikarya; Ascomycota; Pezizomycotina; Sordariomycetes; Hypocreomycetidae; Glomerellales; Glomerellaceae; Colletotrichum sublineola [KDN60298.1]
Eukaryota; Fungi; Dikarya; Ascomycota; Pezizomycotina; Sordariomycetes; Hypocreomycetidae; Glomerellales; Glomerellaceae; Colletotrichum tofieldiae [KZL71654.1]
Eukaryota; Fungi; Dikarya; Ascomycota; Pezizomycotina; Sordariomycetes; Hypocreomycetidae; Glomerellales; Plectosphaerellaceae; Verticillium alfalfae VaMs.102 [XP_003009239.1 XP_003003053.1]
Eukaryota; Fungi; Dikarya; Ascomycota; Pezizomycotina; Sordariomycetes; Hypocreomycetidae; Glomerellales; Plectosphaerellaceae; Verticillium dahliae VdLs.17 [XP_009653360.1 XP_009650204.1]
Eukaryota; Fungi; Dikarya; Ascomycota; Pezizomycotina; Sordariomycetes; Hypocreomycetidae; Glomerellales; Plectosphaerellaceae; Verticillium longisporum [CRK26008.1 CRK17720.1 CRK33601.1 CRK21680.1 CRK27175.1]
Eukaryota; Fungi; Dikarya; Ascomycota; Pezizomycotina; Sordariomycetes; Hypocreomycetidae; Hypocreales; Clavicipitaceae; Aschersonia aleyrodis RCEF 2490 [KZZ98426.1]
Eukaryota; Fungi; Dikarya; Ascomycota; Pezizomycotina; Sordariomycetes; Hypocreomycetidae; Hypocreales; Clavicipitaceae; Metarhizium acridum CQMa 102 [XP_007806607.1]

Eukaryota; Fungi; Dikarya; Ascomycota; Pezizomycotina; Sordariomycetes; Hypocreomycetidae; Hypocreales; Clavicipitaceae; Metarhizium album ARSEF 1941 [KHN96202.1]
Eukaryota; Fungi; Dikarya; Ascomycota; Pezizomycotina; Sordariomycetes; Hypocreomycetidae; Hypocreales; Clavicipitaceae; Metarhizium anisopliae [KFG77837.1]
Eukaryota; Fungi; Dikarya; Ascomycota; Pezizomycotina; Sordariomycetes; Hypocreomycetidae; Hypocreales; Clavicipitaceae; Metarhizium anisopliae BRIP 53293 [KJK82320.1]
Eukaryota; Fungi; Dikarya; Ascomycota; Pezizomycotina; Sordariomycetes; Hypocreomycetidae; Hypocreales; Clavicipitaceae; Metarhizium brunneum ARSEF 3297 [XP_014540615.1]
Eukaryota; Fungi; Dikarya; Ascomycota; Pezizomycotina; Sordariomycetes; Hypocreomycetidae; Hypocreales; Clavicipitaceae; Metarhizium guizhouense ARSEF 977 [KID88983.1]
Eukaryota; Fungi; Dikarya; Ascomycota; Pezizomycotina; Sordariomycetes; Hypocreomycetidae; Hypocreales; Clavicipitaceae; Metarhizium robertsii ARSEF 23 [XP_007824393.1]
Eukaryota; Fungi; Dikarya; Ascomycota; Pezizomycotina; Sordariomycetes; Hypocreomycetidae; Hypocreales; Clavicipitaceae; Metarhizium; Metarhizium majus ARSEF 297 [XP_014576177.1]
Eukaryota; Fungi; Dikarya; Ascomycota; Pezizomycotina; Sordariomycetes; Hypocreomycetidae; Hypocreales; Clavicipitaceae; Pochonia chlamydosporia 170 [XP_018142434.1]
Eukaryota; Fungi; Dikarya; Ascomycota; Pezizomycotina; Sordariomycetes; Hypocreomycetidae; Hypocreales; Cordycipitaceae; Beauveria bassiana ARSEF 2860 [XP_008600218.1 XP_008596320.1]
Eukaryota; Fungi; Dikarya; Ascomycota; Pezizomycotina; Sordariomycetes; Hypocreomycetidae; Hypocreales; Cordycipitaceae; Beauveria bassiana D1-5 [KGQ07807.1 KGQ09312.1]
Eukaryota; Fungi; Dikarya; Ascomycota; Pezizomycotina; Sordariomycetes; Hypocreomycetidae; Hypocreales; Cordycipitaceae; Cordyceps confragosa [OAR01609.1]
Eukaryota; Fungi; Dikarya; Ascomycota; Pezizomycotina; Sordariomycetes; Hypocreomycetidae; Hypocreales; Cordycipitaceae; Cordyceps confragosa RCEF 1005 [OAA64114.1]
Eukaryota; Fungi; Dikarya; Ascomycota; Pezizomycotina; Sordariomycetes; Hypocreomycetidae; Hypocreales; Cordycipitaceae; Cordyceps militaris CM01 [XP_006670172.1]
Eukaryota; Fungi; Dikarya; Ascomycota; Pezizomycotina; Sordariomycetes; Hypocreomycetidae; Hypocreales; Cordycipitaceae; Cordyceps; Cordyceps brongniartii RCEF 3172 [OAA37527.1 OAA52846.1]
Eukaryota; Fungi; Dikarya; Ascomycota; Pezizomycotina; Sordariomycetes; Hypocreomycetidae; Hypocreales; Cordycipitaceae; Isaria fumosorosea ARSEF 2679 [XP_018699788.1]
Eukaryota; Fungi; Dikarya; Ascomycota; Pezizomycotina; Sordariomycetes; Hypocreomycetidae; Hypocreales; Hypocreaceae; Trichoderma atroviride IMI 206040 [XP_013946939.1]
Eukaryota; Fungi; Dikarya; Ascomycota; Pezizomycotina; Sordariomycetes; Hypocreomycetidae; Hypocreales; Hypocreaceae; Trichoderma gamsii [XP_018659236.1]
Eukaryota; Fungi; Dikarya; Ascomycota; Pezizomycotina; Sordariomycetes; Hypocreomycetidae; Hypocreales; Hypocreaceae; Trichoderma guizhouense [OPB45648.1 OPB46051.1 OPB45515.1]
Eukaryota; Fungi; Dikarya; Ascomycota; Pezizomycotina; Sordariomycetes; Hypocreomycetidae; Hypocreales; Hypocreaceae; Trichoderma harzianum [KKO97931.1 KKP02980.1 KKO96688.1]
Eukaryota; Fungi; Dikarya; Ascomycota; Pezizomycotina; Sordariomycetes; Hypocreomycetidae; Hypocreales; Hypocreaceae; Trichoderma parareesei [OTA03272.1]
Eukaryota; Fungi; Dikarya; Ascomycota; Pezizomycotina; Sordariomycetes; Hypocreomycetidae; Hypocreales; Hypocreaceae; Trichoderma reesei QM6a [XP_006969233.1 XP_006969192.1]
Eukaryota; Fungi; Dikarya; Ascomycota; Pezizomycotina; Sordariomycetes; Hypocreomycetidae; Hypocreales; Hypocreaceae; Trichoderma reesei RUT C-30 [ETR97734.1]
Eukaryota; Fungi; Dikarya; Ascomycota; Pezizomycotina; Sordariomycetes; Hypocreomycetidae; Hypocreales; Hypocreaceae; Trichoderma virens Gv29-8 [XP_013954991.1 XP_013954867.1]
Eukaryota; Fungi; Dikarya; Ascomycota; Pezizomycotina; Sordariomycetes; Hypocreomycetidae; Hypocreales; Nectriaceae; Fusarium graminearum PH-1 [XP_011318167.1]
Eukaryota; Fungi; Dikarya; Ascomycota; Pezizomycotina; Sordariomycetes; Hypocreomycetidae; Hypocreales; Nectriaceae; Fusarium langsethiae [KPA41043.1]

Eukaryota; Fungi; Dikarya; Ascomycota; Pezizomycotina; Sordariomycetes; Hypocreomycetidae; Hypocreales; Nectriaceae; Fusarium poae [OBS28331.1]
Eukaryota; Fungi; Dikarya; Ascomycota; Pezizomycotina; Sordariomycetes; Hypocreomycetidae; Hypocreales; Nectriaceae; Fusarium pseudograminearum CS3096 [XP_009251677.1]
Eukaryota; Fungi; Dikarya; Ascomycota; Pezizomycotina; Sordariomycetes; Hypocreomycetidae; Hypocreales; Nectriaceae; Fusarium; Fusarium avenaceum [KIL93869.1 KIL86417.1 KIL92275.1 KIL88325.1]
Eukaryota; Fungi; Dikarya; Ascomycota; Pezizomycotina; Sordariomycetes; Hypocreomycetidae; Hypocreales; Nectriaceae; Fusarium; Fusarium fujikuroi [KLP05755.1 KLO94714.1 KLP09798.1 KLP02364.1 KLO86373.1]
Eukaryota; Fungi; Dikarya; Ascomycota; Pezizomycotina; Sordariomycetes; Hypocreomycetidae; Hypocreales; Nectriaceae; Fusarium; Fusarium fujikuroi IMI 58289 [CCT70677.1 CCT61441.1]
Eukaryota; Fungi; Dikarya; Ascomycota; Pezizomycotina; Sordariomycetes; Hypocreomycetidae; Hypocreales; Nectriaceae; Fusarium; Fusarium oxysporum FOSC 3-a [EWY87714.1]
Eukaryota; Fungi; Dikarya; Ascomycota; Pezizomycotina; Sordariomycetes; Hypocreomycetidae; Hypocreales; Nectriaceae; Fusarium; Fusarium oxysporum Fo47 [EWZ40807.1]
Eukaryota; Fungi; Dikarya; Ascomycota; Pezizomycotina; Sordariomycetes; Hypocreomycetidae; Hypocreales; Nectriaceae; Fusarium; Fusarium oxysporum Fo5176 [EGU82021.1 EGU85310.1 EGU72432.1]
Eukaryota; Fungi; Dikarya; Ascomycota; Pezizomycotina; Sordariomycetes; Hypocreomycetidae; Hypocreales; Nectriaceae; Fusarium; Fusarium oxysporum f. sp. conglutinans race 2 54008 [EXL70537.1 EXL66200.1]
Eukaryota; Fungi; Dikarya; Ascomycota; Pezizomycotina; Sordariomycetes; Hypocreomycetidae; Hypocreales; Nectriaceae; Fusarium; Fusarium oxysporum f. sp. cubense race 1 [ENH65723.1]
Eukaryota; Fungi; Dikarya; Ascomycota; Pezizomycotina; Sordariomycetes; Hypocreomycetidae; Hypocreales; Nectriaceae; Fusarium; Fusarium oxysporum f. sp. cubense tropical race 4 54006 [EXM08571.1 EXL91734.1]
Eukaryota; Fungi; Dikarya; Ascomycota; Pezizomycotina; Sordariomycetes; Hypocreomycetidae; Hypocreales; Nectriaceae; Fusarium; Fusarium oxysporum f. sp. lycopersici 4287 [XP_018236387.1 XP_018248779.1]
Eukaryota; Fungi; Dikarya; Ascomycota; Pezizomycotina; Sordariomycetes; Hypocreomycetidae; Hypocreales; Nectriaceae; Fusarium; Fusarium oxysporum f. sp. lycopersici MN25 [EWZ89447.1]
Eukaryota; Fungi; Dikarya; Ascomycota; Pezizomycotina; Sordariomycetes; Hypocreomycetidae; Hypocreales; Nectriaceae; Fusarium; Fusarium oxysporum f. sp. melonis 26406 [EXK33498.1 EXK26078.1 EXK46349.1]
Eukaryota; Fungi; Dikarya; Ascomycota; Pezizomycotina; Sordariomycetes; Hypocreomycetidae; Hypocreales; Nectriaceae; Fusarium; Fusarium oxysporum f. sp. pisi HDV247 [EXA41580.1 EXA54760.1]
Eukaryota; Fungi; Dikarya; Ascomycota; Pezizomycotina; Sordariomycetes; Hypocreomycetidae; Hypocreales; Nectriaceae; Fusarium; Fusarium oxysporum f. sp. radicis-lycopersici 26381 [EXL44275.1]
Eukaryota; Fungi; Dikarya; Ascomycota; Pezizomycotina; Sordariomycetes; Hypocreomycetidae; Hypocreales; Nectriaceae; Fusarium; Fusarium oxysporum f. sp. raphani 54005 [EXK79905.1]
Eukaryota; Fungi; Dikarya; Ascomycota; Pezizomycotina; Sordariomycetes; Hypocreomycetidae; Hypocreales; Nectriaceae; Fusarium; Fusarium oxysporum f. sp. vasinfectum 25433 [EXM14855.1]
Eukaryota; Fungi; Dikarya; Ascomycota; Pezizomycotina; Sordariomycetes; Hypocreomycetidae; Hypocreales; Nectriaceae; Fusarium; Fusarium proliferatum [CVK92246.1 CVL03287.1]
Eukaryota; Fungi; Dikarya; Ascomycota; Pezizomycotina; Sordariomycetes; Hypocreomycetidae; Hypocreales; Nectriaceae; Fusarium; Fusarium proliferatum ET1 [CZR35828.1]
Eukaryota; Fungi; Dikarya; Ascomycota; Pezizomycotina; Sordariomycetes; Hypocreomycetidae; Hypocreales; Nectriaceae; Fusarium; Fusarium verticillioides 7600 [XP_018744479.1 XP_018756998.1]
Eukaryota; Fungi; Dikarya; Ascomycota; Pezizomycotina; Sordariomycetes; Hypocreomycetidae; Hypocreales; Nectriaceae; Fusarium; Nectria haematococca mpVI 77-13-4 [XP_003041658.1 XP_003041870.1 XP_003043242.1]
Eukaryota; Fungi; Dikarya; Ascomycota; Pezizomycotina; Sordariomycetes; Hypocreomycetidae; Hypocreales; Nectriaceae; Neonectria ditissima [KPM37551.1 KPM46313.1]
Eukaryota; Fungi; Dikarya; Ascomycota; Pezizomycotina; Sordariomycetes; Hypocreomycetidae; Hypocreales; Ophiocordycipitaceae; Hirsutella minnesotensis 3608 [KJZ72762.1 KJZ73950.1 KJZ75503.1]
Eukaryota; Fungi; Dikarya; Ascomycota; Pezizomycotina; Sordariomycetes; Hypocreomycetidae; Hypocreales; Ophiocordycipitaceae; Purpureocillium lilacinum [OAQ67448.1 XP_018178455.1]

Eukaryota; Fungi; Dikarya; Ascomycota; Pezizomycotina; Sordariomycetes; Sordariomycetidae; Diaporthales; Diaporthaceae; Diaporthe helianthi [OCW39740.1]
Eukaryota; Fungi; Dikarya; Ascomycota; Pezizomycotina; Sordariomycetes; Sordariomycetidae; Magnaporthales; Magnaporthaceae; Gaeumannomyces tritici R3-111a-1 [XP_009216551.1 XP_009219164.1]
Eukaryota; Fungi; Dikarya; Ascomycota; Pezizomycotina; Sordariomycetes; Sordariomycetidae; Magnaporthales; Magnaporthaceae; Magnaporthe oryzae 70-15 [XP_003717446.1 XP_003710359.1]
Eukaryota; Fungi; Dikarya; Ascomycota; Pezizomycotina; Sordariomycetes; Sordariomycetidae; Magnaporthales; Magnaporthaceae; Magnaporthiopsis poae ATCC 64411 [KLU88346.1 KLU82625.1]
Eukaryota; Fungi; Dikarya; Ascomycota; Pezizomycotina; Sordariomycetes; Xylariomycetidae; Xylariales; Diatrypaceae; Eutypa lata UCREL1 [XP_007796055.1]
Eukaryota; Fungi; Dikarya; Ascomycota; Pezizomycotina; Sordariomycetes; Xylariomycetidae; Xylariales; Sporocadaceae; Pestalotiopsis fici W106-1 [XP_007831089.1]
Eukaryota; Fungi; Dikarya; Ascomycota; Pezizomycotina; Sordariomycetes; Xylariomycetidae; Xylariales; Xylariaceae; Rosellinia necatrix [GAP92155.1]
Eukaryota; Fungi; Dikarya; Basidiomycota; Agaricomycotina; Agaricomycetes; Agaricomycetidae; Agaricales; Cortinariaceae; Hebeloma cylindrosporum h7 [KIM41776.1]
Eukaryota; Fungi; Dikarya; Basidiomycota; Agaricomycotina; Agaricomycetes; Agaricomycetidae; Agaricales; Physalacriaceae; Cylindrobasidium torrendii FP15055 ss-10 [KIY64279.1]
Eukaryota; Fungi; Dikarya; Basidiomycota; Agaricomycotina; Agaricomycetes; Agaricomycetidae; Agaricales; Strophariaceae; Galerina marginata CBS 339.88 [KDR78349.1]
Eukaryota; Fungi; Dikarya; Basidiomycota; Agaricomycotina; Agaricomycetes; Agaricomycetidae; Agaricales; Strophariaceae; Hypholoma sublateritium FD-334 SS-4 [KJA17847.1 KJA17849.1 KJA17848.1]
Eukaryota; Fungi; Dikarya; Basidiomycota; Agaricomycotina; Agaricomycetes; Agaricomycetidae; Agaricales; Tricholomataceae; Laccaria amethystina LaAM-08-1 [KIK09601.1 KIK09600.1]
Eukaryota; Fungi; Dikarya; Basidiomycota; Agaricomycotina; Agaricomycetes; Agaricomycetidae; Agaricales; Tricholomataceae; Laccaria bicolor S238N-H82 [XP_001879974.1 XP_001891237.1 XP_001879973.1 XP_001891238.1]
Eukaryota; Fungi; Dikarya; Basidiomycota; Agaricomycotina; Agaricomycetes; Phallomycetidae; Geastrales; Sphaerobolaceae; Sphaerobolus stellatus SS14 [KIJ33480.1]
Eukaryota; Fungi; Zoopagomycota; Entomophthoromycotina; Basidiobolomycetes; Basidiobolales; Basidiobolaceae; Basidiobolus meristosporus CBS 931.73 [ORX88910.1 ORX91286.1 ORX98517.1]
Eukaryota; Fungi; Zoopagomycota; Kickxellomycotina; Harpellales; Legeriomycetaceae; Smittium culicis [OMJ19911.1 OMJ16329.1 OMJ25745.1 OMJ20071.1 OMJ18476.1 OMJ19929.1 OMJ07914.1]
Eukaryota; Fungi; Zoopagomycota; Kickxellomycotina; Harpellales; Legeriomycetaceae; Smittium mucronatum [OLY82477.1 OLY84473.1]
Eukaryota; Fungi; Zoopagomycota; Kickxellomycotina; Harpellales; Legeriomycetaceae; Zancudomyces culisetae [OMH82354.1]
Eukaryota; Metazoa; Ecdysozoa; Arthropoda; Hexapoda; Collembola; Collembola; Entomobryomorpha; Isotomoidea; Isotomidae; Proisotominae; Folsomia candida [OXA61332.1 OXA62220.1 OXA63930.1 OXA45249.1 OXA60469.1 OXA60075.1]
Eukaryota; Opisthokonta; Metazoa; Eumetazoa; Bilateria; Protostomia; Ecdysozoa; Nematoda; Chromadorea; Tylenchida; Tylenchomorpha; Tylenchoidea; Heteroderidae; Heteroderinae; Heterodera; Heterodera avenae [AVA09682.1]
Eukaryota; Stramenopiles; Oomycetes; Peronosporales; Phytophthora infestans T30-4 [XP_002906333.1 XP_002906272.1 XP_002906332.1]
Eukaryota; Stramenopiles; Oomycetes; Peronosporales; Phytophthora megakarya [OWZ11341.1 OWZ22689.1 OWZ15571.1 OWY94368.1]
Eukaryota; Stramenopiles; Oomycetes; Peronosporales; Phytophthora nicotianae [KUF98507.1 KUF91726.1]
Eukaryota; Stramenopiles; Oomycetes; Peronosporales; Phytophthora parasitica [ETL89750.1 ETK77788.1 ETM37609.1 ETL31176.1 ETK77741.1 ETL84433.1 ETM37653.1 ETL84477.1 ETK77785.1 ETL31216.1]
Eukaryota; Stramenopiles; Oomycetes; Peronosporales; Phytophthora parasitica CJ01A1 [ETP07429.1 ETP07431.1 ETP23127.1 ETP07428.1]
Eukaryota; Stramenopiles; Oomycetes; Peronosporales; Phytophthora parasitica INRA-310 [XP_008911684.1 XP_008911682.1 XP_008911683.1 XP_008911681.1 XP_008911680.1 XP_008911696.1]
Eukaryota; Stramenopiles; Oomycetes; Peronosporales; Phytophthora parasitica P10297 [ETP35493.1 ETP35492.1 ETP35510.1 ETP35489.1]
Eukaryota; Stramenopiles; Oomycetes; Peronosporales; Phytophthora parasitica P1569 [ETI37551.1 ETI43101.1 ETI37569.1]
Eukaryota; Stramenopiles; Oomycetes; Peronosporales; Phytophthora parasitica P1976 [ETO66338.1 ETO66336.1 ETO66335.1]

Eukaryota; Stramenopiles; Oomycetes; Peronosporales; Phytophthora sojae [XP_009515363.1 XP_009515362.1 XP_009515359.1 XP_009515360.1 XP_009515350.1 XP_009525378.1 XP_009515361.1 XP_009522151.1 XP_009515355.1 XP_009515351.1]
Eukaryota; Stramenopiles; Oomycetes; Saprolegniales; Saprolegniaceae; Achlya hypogyna [AIG56085.1 OQR88575.1]
Eukaryota; Stramenopiles; Oomycetes; Saprolegniales; Saprolegniaceae; Aphanomyces astaci [XP_009834932.1]
Eukaryota; Stramenopiles; Oomycetes; Saprolegniales; Saprolegniaceae; Saprolegnia diclina VS20 [XP_008619259.1 XP_008612284.1 XP_008620304.1 XP_008620098.1]
Eukaryota; Stramenopiles; Oomycetes; Saprolegniales; Saprolegniaceae; Saprolegnia parasitica CBS 223.65 [XP_012203825.1 XP_012209728.1 XP_012206248.1]
